# SARS-CoV-2 and the DNA damage response

**DOI:** 10.1099/jgv.0.001918

**Published:** 2023-11-10

**Authors:** Roger J. Grand

**Affiliations:** ^1^​ Institute for Cancer and Genomic Science, The Medical School, University of Birmingham, Birmingham, UK

**Keywords:** SARS-CoV-2, COVID-19, DNA repair, DNA damage response, senescence

## Abstract

The recent coronavirus disease 2019 (COVID-19) pandemic was caused by severe acute respiratory syndrome coronavirus 2 (SARS-CoV-2). COVID-19 is characterized by respiratory distress, multiorgan dysfunction and, in some cases, death. The virus is also responsible for post-COVID-19 condition (commonly referred to as ‘long COVID’). SARS-CoV-2 is a single-stranded, positive-sense RNA virus with a genome of approximately 30 kb, which encodes 26 proteins. It has been reported to affect multiple pathways in infected cells, resulting, in many cases, in the induction of a ‘cytokine storm’ and cellular senescence. Perhaps because it is an RNA virus, replicating largely in the cytoplasm, the effect of SARS-Cov-2 on genome stability and DNA damage responses (DDRs) has received relatively little attention. However, it is now becoming clear that the virus causes damage to cellular DNA, as shown by the presence of micronuclei, DNA repair foci and increased comet tails in infected cells. This review considers recent evidence indicating how SARS-CoV-2 causes genome instability, deregulates the cell cycle and targets specific components of DDR pathways. The significance of the virus’s ability to cause cellular senescence is also considered, as are the implications of genome instability for patients suffering from long COVID.

## Introduction

By mid-October 2023 over three-quarters of a billion cases of coronavirus disease 2019 (COVID-19) had been reported, with almost seven million deaths, worldwide [WHO Coronavirus (COVID-19) Dashboard]. Approximately half of COVID-19 survivors, regardless of hospitalization status, still experience a range of symptoms after 4 to 6 months, which has been termed ‘long COVID’ [1–7]. Prolonged symptoms include immune dysregulation, vascular and circulatory problems, respiratory and gastrointestinal system problems, neurological problems and general fatigue, with symptoms resembling myalgic encephalaomyelitis/chronic fatigue syndrome (ME/CFS) (reviewed, for example, in [7]). COVID-19 is caused by infection with severe acute respiratory syndrome coronavirus-2 (SARS-CoV-2) [8–10]. SARS-CoV-2, like other coronaviruses, is a member of the order Nidovirales and the family *Coronaviridae*. It is closely related to other betacoronaviruses – severe acute respiratory syndrome coronavirus (SARS-CoV) and Middle East respiratory syndrome coronavirus (MERS-CoV), with approximately 79 and 50% homology, respectively, at the nucleotide level [8, 11].

SARS-CoV-2 has a single-stranded, positive-sense RNA genome of approximately 30 kb. Following infection, two open reading frames, ORF1a and ORF1b, encompassing approximately two-thirds of the viral genome at the 5′ end, are translated into two polyproteins (pp1a and pp1ab), while four structural proteins (spike, envelope, membrane and nucleocapsid; S, E, M, N, respectively) and a number of accessory proteins (ORF3a, ORF3b, ORF6, ORF7a, ORF7b, ORF8, ORF9b and ORF14) are encoded by the 3′ third of the genome [12–17]. The polyproteins are cleaved by viral proteases, encoded by Nsp3 and Nsp5, to give 16 non-structural proteins (Nsp1–16) [18–21].

Infection with SARS-CoV-2 leads to dysregulation of many pathways in the host cell, most notably those involved with the immune response and inflammation, oxidative stress, RNA metabolism, homeostasis, cell cycle regulation, senescence, autophagy and apoptosis. Up to now, relatively little attention had been paid to the relationship between the virus and genome stability and the DNA damage response (DDR). However, it is now becoming clear that SARS-CoV-2 causes damage to the host cell DNA and has a complex interaction with the cellular DDR. In this review I have attempted to summarize published data relevant to this relationship and have gone on to consider what the implications might be for patients suffering from long COVID. Because almost all the information discussed here has been published recently, and may be preliminary in a few instances, in some cases there are inconsistencies and divergent views, which will need further evaluation for verification in future; however, it is hoped that the case has been made that the DDR is an important target for SARS-CoV-2.

## Viruses and the DNA damage response

The cellular DDR comprises a series of complex, sometimes overlapping, pathways that can detect and repair different forms of damage to the cellular genome. At least seven pathways can be distinguished. For example, double-strand breaks (DSBs), arising as a result of exogenous or endogenous factors, are repaired either by non-homologous end joining (NHEJ) or homologous recombination (HR) [22–28]. HR is an error-free repair mechanism, occurring during the S and G2 phases of the cell cycle, which relies on the presence of the sister chromatid as a repair template. NHEJ occurs throughout the cell cycle and may, very rarely, introduce errors into the repaired DNA. Mismatch repair (MMR) is primarily for the repair of base–base mismatches and insertion/deletion mispairs generated during DNA replication and recombination [29–32]. The base excision repair (BER) pathway repairs most endogenous base lesions and abnormal bases in the genome and is also involved in repair of DNA single-strand breaks [33, 34]. Nucleotide excision repair (NER) is the main pathway used by mammals to remove bulky DNA adducts, which disrupt the structure of the DNA double helix, such as those formed by UV radiation [35, 36]. Inter-strand crosslink repair (ICL) fixes crosslinks formed between the two DNA strands. Inter-strand crosslinks are repaired by the Fanconi anaemia (FA) pathway, whereas intra-strand crosslinks are repaired by the NER pathway [37–39]. There is also a direct repair pathway that is dependent on the ability of O^6^-methylguanine-DNA methyltransferase (MGMT) to remove alkyl groups from the O^6^ position on guanine [40, 41]. These pathways, in the context of viral infection, have been described in more detail in several reviews over the past decade (for example [42–44]).

All DNA viruses seem to impinge on one or more of these repair pathways during infection, either activating or antagonizing them [42, 45–48]. Initial infection by DNA viruses generally results in activation of phosphatidylinositol 3-kinase-related kinases (PIKKs), resulting in phosphorylation of downstream targets such as γH2AX and the Chk1 and Chk2 kinases. This activation may be due to the presence of viral DNA and/or expression of viral proteins that affect host cell processes such as transcription [47, 48]. The viruses then go on to utilize or inactivate, depending on the virus in question, differing components of the repair pathways to facilitate viral replication. Thus, for example, some viruses, such as adenovirus (HAdV) and herpes simplex virus 1 (HSV1), inactivate double-strand break repair by targeted degradation of MRE11, BLM and DNA Ligase IV (by HAdVs, depending on the type) or RNF8 and DNA-PKcs, (by HSV1) [49–52]. On the other hand, most gammaherpesviruses require ATM for lytic replication [53–57], as do polyoma viruses [58, 59]. Regardless of whether a DNA virus activates or antagonizes the DDR they all recruit damage response proteins to their viral replication centres (VRCs) (see, for example, [55, 60–65]). It is assumed that their function there is to facilitate the production and integrity of viral DNA, although direct evidence for this is limited.

Our knowledge of the relationship of RNA viruses to the DDR pathways is much more limited (reviewed in [43]). The mode of replication of RNA viruses is governed largely by the configuration of their genomes. Coronaviruses, such as SARS-CoV-2, have a positive-sense, single-stranded genome that serves as mRNA and is translated in the cytoplasm largely into a polyprotein before its cleavage, giving rise to the replicase–transcriptase complex. This complex synthesizes a negative-strand RNA that acts as a template for transcription of genomic RNA and multiple subgenomic mRNAs in the infected cell. Membrane-associated VRCs, forming in the cytoplasm and containing viral proteins, are sites for the production of new viral RNA [66, 67]. (For a detailed description of the replication of RNA viruses in general see, for example, various chapters in [68]).

As most RNA viruses largely replicate in the cytoplasm, it might be expected that they have relatively little interaction with the cellular DDR. However, this is not the case and representative viruses from many families have been shown to cause genome instability and activation of DDR pathways (reviewed in [43]). For example, Chikungunya and Sindbis viruses – members of the family *Togaviridae* that, like SARS-CoV-2 and other coronaviruses, replicate in the cytoplasm – activate ATR and PARP1 pathways following infection [69, 70]. Other RNA viruses, such as human immunodeficiency virus-1 (HIV-1) (a member of the family *Retroviridae*) and influenza A virus (IAV) (a member of the family *Orthomyxoviridae*), replicate partially in the nucleus and so might be expected to be more likely to cause DNA damage. HIV-1 causes double-strand breaks as a result of binding of its VPR protein to chromatin [71]. Vpr expression also activates ATM and leads to the formation of repair foci [72]. IAV infection leads to DNA damage and formation of γH2AX repair foci [73, 74]. Thus, it appears that the conformation of the viral genome, positive or negative strand, or even location of replication, has little influence over whether an RNA virus species impacts on the host cell DDR. The explosion of scientific interest in coronaviruses, and SARS-CoV-2 in particular, triggered by the COVID-19 pandemic, has meant that our understanding of the relationship of this RNA virus to the DDR has increased appreciably. In this review I discuss these latest observations on the mechanisms by which SARS-CoV-2 can induce DNA damage and activate or inhibit DNA repair pathways. I also consider the implications of this for the long Covid syndrome. Previous reviews have also examined the relationship between SARS-CoV-2 and the DDR [75, 76]. In the latter case, emphasis has been placed on the host immune system.

## SARS-COV-2

SARS-CoV-2, like SARS-CoV, gains entry to the host cell through association with cellular attachment factors, such as glycans and integrins, and binding of the spike (S) glycoprotein to angiotensin-converting enzyme 2 (ACE2) [77–81]. S is cleaved to give the functionally distinct S1 and S2 subunits by a cellular protease [79, 82]. The incorporation of the basic cleavage site (PRRAR) at the S1/S2 boundary facilitates the cleavage reaction, resulting in enhanced infection [82]. An additional cleavage site is present in S2 [79]. S1 contains the receptor-binding domain, whereas the S2 transmembrane domain mediates fusion of the viral and cellular membranes, leading to syncytial formation. Cleavage of the S protein, predominantly by transmembrane protease serine 2 (TMPRSS2), is necessary for fusion and the viral entry to occur [79, 81, 82].

The viral genomic RNA is then released into the host cell cytoplasm, initiating viral gene expression (reviewed, for example, in [12]). Initially, the SARS-CoV-2 genome is copied to produce a full-length negative version that is a template for the production of new positive-sense genomic RNA, which can be translated into proteins or used as genomes in progeny viruses. As with other members of the order Nidovirales, transcription is discontinuous, such that a set of 3′ to 5′ co-terminal sub-genomic RNAs are produced. These are used to synthesize negative-strand RNAs, which are then used as templates to synthesize positive-sense mRNAs, which, in turn, are translated to give structural and accessory proteins [12, 83–86].

As mentioned above, ORF1a and ORF1b are translated from the genome, giving rise to the polyproteins pp1a and pp1ab [83, 84]. Cleavage of these by viral cysteine proteases, located in Nsp3 and Nsp5, produces Nsps 1–11 from pp1a and Nsps 12–16 from pp1ab [12–14, 87–89]. Rapid proteolytic release of Nsp1 leads to inhibition of translation of certain host cell proteins [90–92]. Nsps 2–16 form the viral replication/transcription complex (RTC), with Nsps 12–16 providing the enzymes necessary for RNA synthesis, modification and proofreading. The remaining Nsps (2–11) appear to play slightly subsidiary roles, supporting replication (reviewed in [12, 93, 94]). Nsp 12 is the RNA-dependent RNA polymerase (RdRP), and Np7 and Nsp 8 act as co-factors [95, 96]. Nsp7 and Nsp 8 have been suggested to possess primase activity [97]. Nsp14 has proofreading capability and has both 3′−5′ exonuclease and guanine N7 methyltransferase activity [98–100]. The N7 methyltransferase activity is required for 5′ capping of viral RNAs, which protects them from host anti-viral defences [95–98]. Nsp10 associates with Nsp14 and enhances its exonuclease activity [98–100]. Nsp13 is an RNA helicase and is essential for transcription and translation [101, 102]. Nsp16 is a 2′-O-methyltransferase, whilst Nsp15 has endoribonuclease activity [103–106] (these and other functions, with additional relevant references, are summarized in Table 1).

**Table 1.** SARS-CoV-2 proteins, summary of their subcellular localization and their function. A very brief description of the function is given, together with appropriate references

SARS-CoV-2 protein	Tentative subcellular location	Functions	References
Nsp1	Cytoplasmic	Induces host cell RNA cleavage, host shut off, inhibits host RNA translation and IFN response. Inhibits STAT1 phosphorylation	[90–92, 253–255]
Nsp2	Cytoplasmic	Impairs IFN production, represses mRNA translation	[256, 257]
Nsp3	Cytoplasmic	Protease activity (polyprotein processing). Involved in generation of DMVs with Nsp4. Interacts with N	[87, 258–260]
Nsp4	ER, cytoplasmic	DMV formation with Nsp3	[260]
Nsp5	Cytoplasmic	Protease activity – 3CL^pro^ and M^pro^ (polyprotein processing). Counteracts RIG-I MAVS signalling and IFN signalling	[20, 21, 88, 89, 261]
Nsp6	Golgi, ER	DMV formation. Replication organelle with Nsp3, Nsp4. ER membrane zippering	[260, 262]
Nsp7	Cytoplasmic	Accessory protein for RNA polymerase. Inhibits IFN production by targeting RIG-I	[96, 263–265]
Nsp8	Cytoplasmic	Primase. Accessory protein for RNA polymerase. Interacts with MDA-5	[96, 97, 263, 264, 266]
Nsp9	Cytoplasmic, nucleus	RNA binding protein. Associates with Nsp12. Recruits protein for 5′ capping. Binds nuclear pore complex proteins	[267–269]
Nsp10	Cytoplasmic, present in vesicular structures	Co-factor for Nsp14 and Nsp16 methyl transferases. Component of exonuclease complex	[98, 99, 105, 270, 271]
Nsp11	Cytoplasmic	Short peptide of unknown function	[272]
Nsp12	Cytoplasmic, Golgi	RNA-dependent RNA polymerase – associates with Nsp7 and Nsp8. Also complex with Nsp14 and Nsp16	[93, 94, 263, 264, 267, 273]
Nsp13	Cytoplasmic	RNA helicase, 5′ triphosphatase. IFN antagonist	[101, 102, 264, 274, 275]
Nsp14	Nucleus	3′−5′ exoribonuclease, N7-methyl transferase. Interacts with RdRP complex. IFN antagonist	[98, 99, 105, 274, 276, 277]
Nsp15	Diffuse; cytoplasmic, Golgi	Endoribonuclease. IFN antagonist	[103, 104, 274, 278]
Nsp16	Endosomes, cytoplasmic	2′-O-methyltransferase. Protects from MDA5 immune response	[105, 106, 279, 280]
Envelope protein (E)	ER, Golgi	Virion structure. Activates TLR2 pathway	[120, 121, 281]
Membrane protein (M)	ER, Golgi, endosomal, cytoplasmic	Virion structure. IFN antagonist. Binds PCNA and N protein	[107–109]
Nucleocapsid (N)	Diffuse cytoplasmic	Houses viral genome, virus particle release. Counteracts RIG-I signalling	[107, 113–115, 282, 283]
Spike protein (S)	ER, plasma membrane, cytoplasmic	Viral entry, binds host cell receptor	[77–82, 117, 118, 284, 285]
ORF3a	ER, Golgi, endosomal	Viroporin, also promotes virus release. Induces apoptosis. Suppresses INF signalling	[125, 126, 286–292]
ORF3b	ER, Golgi, endosomal	IFN antagonist	[293]
ORF6	ER, nuclear membrane, Golgi, membrane vesicles, cytoplasm	IFN antagonist	[109, 126, 294–296]
ORF7a	ER, Golgi	IFN antagonist, inhibits IRSE promoter activity	[126, 287, 297–299]
ORF7b	ER, Golgi, cytoplasmic	IFN antagonist, inhibits IRSE promoter activity	[126, 297]
ORF8	ER, Golgi, also secreted	IFN antagonist. Interacts with MHC-I and mediates its degradation	[126, 288, 300–303]
ORF9b	Diffuse, mitochondrial membrane	Compromising the mitochondria, binds TOM70. Affects IFN synthesis. Antagonizes IFN signalling. Interacts with host cell organelles	[126, 304–309]
ORF9c	Nuclear membrane, cytoplasmic	Affects lipid remodelling and ER stress response. Interacts with host cell organelles	[126, 310]
ORF10	ER, Golgi, cytoplasmic	Inhibits IFN signalling by inhibiting MAVS expression	[126, 311]

DMV, Double membrane vesicle.; RdRP, RNA dependent RNA polymerase.

The SARS-CoV-2 membrane protein (M) is the most abundant structural protein and is important for virus assembly and determining virus morphology and membrane budding [107–111]. M protein localizes in the endoplasmic reticulum Golgi intermediate compartment (ERGIC) and is essential for the recruitment of other viral proteins (see Table 1 for a summary of the subcellular distribution of SARS-CoV-2 proteins). It binds to the nucleocapsid (N) protein and together they can form virus-like particles [107]. Although N protein has a structural function, it is also essential for transcription and replication [112]. Through binding to the viral RNA genome, it is involved in the creation of the ribonucleoprotein complexes that regulate replication and viral RNA synthesis. Binding of viral RNA to N protein causes liquid–liquid phase separation; this enhances the interaction of RNA with the RdRP complex, favouring viral replication [112–116].

SARS-CoV-2 spike trimeric glycoprotein (S) is located on the surface of the viral membrane and is responsible for binding to the host cell ACE-2 receptor, as mentioned above [79–82, 117–119]. The envelope (E) protein is an integral membrane protein of low molecular weight (75 aa in SARS-CoV-2) (reviewed in [120, 121]). It is present at low concentration in virus particles but is abundant in infected cells. E protein interacts with the membrane protein (M), helping to maintain the structure of the virus particle. E protein is present inside the virus envelope. In addition, it polymerizes to form a cation-selective ion channel (also termed a viroporin), which is localized in the Golgi, endoplasmic reticulum and ERGIC of the infected cell.

A further set of proteins is encoded on the SARS-CoV-2 genome. These are the accessory proteins, which do not appear to be essential for viral replication but are important for virus–host cell interactions associated with virulence (reviewed in [14, 93, 122–124]) (Table 1). The SARS-CoV-2 accessory proteins ORF6, ORF3b, ORF7a, ORF7b, ORF8, ORF9b and ORF10 are considered to be interferon (IFN) antagonists and play a role in SARS-CoV-2 pathogenesis (reviewed in [125–127]).

## Cell cycle regulation by SARS-COV-2

Following damage to their DNA, eukaryotic cells adopt a number of responses. These include co-ordination of cell cycle progression with DNA repair, as well as chromatin remodelling and transcriptional regulation or cell death. Arrest or delay of cell cycle progression provides time for DNA repair, and this is controlled by a network of signalling pathways that are able to initiate various cell cycle checkpoints (reviewed, for example, in [128, 129]). Simplistically, different checkpoints are activated, depending on where in the cell cycle damage occurs. For example, to prevent entry into S phase with damaged DNA, cells in G1 activate the checkpoint kinases ATM/ATR and Chk1/Chk2. Similarly, the G2/M checkpoint prevents segregation of chromosomes that have incurred unrepaired damage during G2. Depending on which checkpoint is activated or the type of DNA damage, different repair pathways will be activated.

Most viruses take control of the cell cycle of the infected cell to produce an environment conducive to viral replication (reviewed, for example, in [130]). For example, adenoviruses cause progression into a ‘pseudo-S phase’ by the action of E1A proteins that bind RB family members, de-repressing E2F transcription factors, and activating genes required for cell cycling (reviewed in [131, 132]). Similarly, lytic replication of Epstein–Barr virus (EBV) and Kaposi's sarcoma-associated virus (KSHV) leads to S phase accumulation and activation of S phase-specific DNA repair pathways [133, 134]. RNA viruses are also well known to regulate the cell cycle following infection; for example, Rift Valley fever virus (RVFV), a negative-strand RNA virus of the family *Bunyaviridae*, encodes a non-structural (NS) protein that causes S phase arrest by activating the ATM-Chk2 pathway [135]. Several SARS-CoV-2 proteins are involved in the viral regulation of the cell cycle. As will be discussed in detail below, ORF6 and Nsp13 separately cause downregulation of Chk1, leading to cell cycle arrest in S phase [136]. Similarly, SARS-CoV-2 Nsp12 binds to the p50 component of DNA polymerase delta (pol δ) [137]. This interaction, together with a likely association between Nsp13 and the p125 subunit of DNA pol δ, plays a synergistic role in the induction of S phase arrest following co-expression of SARS-CoV-2 Nsp12 and Nsp13 [137]. It has previously been shown that infection of cells with the coronavirus infectious bronchitis virus (IBV) activates the S phase checkpoint and induces cell cycle arrest in S and G2/M phases, partially through the interaction of Nsp13 with the p125 catalytic subunit of pol δ [138]. Contrasting evidence, however, has indicated that the SARS-CoV-2 N protein can cause arrest of kidney cells in G1 by interaction with Smad3, enhancing TGF‐*β*/Smad3 signalling [139]. In further studies it was demonstrated that quercetin can block the interaction, which leads to an inhibition of Smad3 signalling and p21- and p16-dependent G1 cell cycle arrest and cell death *in vivo* and *in vitro* [139, 140]. In studies of SARS-CoV-2, expression of N protein has been shown to cause a cell cycle block in G1/S [141], whereas the SARS-CoV protein blocks S phase progression [142]. In the latter report it was observed that decreased S phase gene expression and decreased phosphorylation of a CDK2 substrate occurred in SARS-CoV-infected cells, which is perhaps more physiologically relevant [142]. However, several other investigations have shown that SARS-CoV 3 a, 3b and 7 a proteins, as well as murine coronavirus infection, cause G0/G1 arrest, and this is required for viral replication (reviewed in [143]).

In a large screen of protein phosphorylation following SARS-CoV-2 infection, kinases whose activity was predicted to be downregulated included several cell cycle kinases (CDK1/2/5 and AURKA) and cell growth-related signalling pathway kinases (PRKACA, AKT1/2, MAPK1/3 and PIM1) [144]. By comparison of public phosphoproteomics datasets, it was concluded that SARS-CoV-2 infection induced a cell cycle block at the S/G2 transition, with relatively few cells in G0, G1 or M [144]. Notably, the observed increase in activity of MAPKp38 is consistent with a cell cycle block [145]. Also, CDK2 T^14^/Y^15^ phosphorylation increased after SARS-CoV-2 infection; this is normally linked to inhibition of progression from G2 phase to M [144, 146, 147]. The presence of the infected cells in S/G2 suggests that any DSBs that occurred would be repaired by homologous recombination, and it is notable that this pathway is activated during SARS-CoV-2 infection [136].

In a wide-ranging survey of transcriptome data obtained following SARS-CoV-2, infection, it was noted that some cell cycle genes were upregulated. These included aurora kinase B (*AURKB*), p21 (*CDKN1A*) and cyclin B1 (*CCNB1*). A limited number of genes were downregulated, such as p57 (CDKN1C) [148]. Overall, it is difficult to reconcile all these results, but it seems reasonable to conclude that SARS-CoV-2 induces infected cells into S phase, facilitating replication.

Because of its multiple roles in cell cycle regulation, DDR, and the induction of apoptosis and autophagy, p53 is a common target for infecting DNA viruses such as adenovirus, human papilloma virus (HPV) and EBV (reviewed in [42, 46]). It can also be a target for RNA viruses; for example, p53 is mislocalized to the cytoplasm by the NS2 protein during hepatitis C virus (HCV) infection [149]. However, only limited information is available on the relationship between SARS-CoV-2 and p53 (reviewed in [150]). It has been shown that the viral protease Nsp5 can act as a repressor of the p53 signalling pathway and that p53 can restrict SARS-CoV-2 production [151]. However, these experiments were carried out in HEK293T cells, where p53 transcriptional activity is compromised. The N protein from another coronavirus [porcine epidemic diarrhoea virus (PEDV)] can induce cell cycle arrest in S phase by direct interaction with p53 in the nucleus [152]. This association has the effect of maintaining high levels of p53, activating the p53-DREAM pathway. Whether such an interaction occurs in SARS-CoV-2-infected cells has not yet been shown. A further relevant interaction has been reported for SARS-CoV Nsp3, which associates with, and stabilizes, RCHY1 (ring finger and CHY zinc finger containing 1, also known as Pirh2) [153]. This has the effect of increasing RCHY1-mediated p53 degradation, presumably affecting cell cycle progression and the DDR [153]. RCHY1 also ubiquitylates the translesion DNA polymerase eta, inhibiting its DNA damage bypass activity, as well as several other significant substrates, such as Chk2, p73 and HDAC2 [154–157]. It has not been unequivocally demonstrated that SARS-CoV-2 Nsp3 binds RCHY1, but the interaction has been shown in a mass spectrometric screen [158] (see below).

## SARS-CoV-2 and DDR

In general, the relationship between viruses and DNA damage and repair can be divided into two aspects. Firstly, damage inflicted on the host cell genome, either directly through viral proteins or indirectly, for example, through the generation of reactive oxygen species (ROS) or the generation of DNA replication stress. Secondly, by affecting host cell DNA damage repair pathways; this can also lead to increased DNA damage as repair to ‘normally occurring’ DNA lesions is compromised. It is clear that SARS-CoV-2 has the potential to induce DNA damage, as evidenced by the presence of numerous micronuclei in syncytia formed in cells expressing S protein and ACE2, as well as activation of the cGAS–STING pathway [159]. Similarly, it has been observed that the DNA of COVID-19 patients shows increased damage compared to healthy controls, as determined by comet assay [136, 160]. Furthermore, in these studies there was a positive correlation between severity of disease, extent of damage and oxidative stress.

### Host cell DNA damage resulting from SARS-CoV-2 infection

Oxidative stress arises as a result of differences between the rate of ROS production and accumulation and the concentration of cellular antioxidants. All coronaviruses affect the cellular redox machinery in favour of viral replication. This also has the effect of increasing inflammation, apoptosis and cellular damage in the host (reviewed in [161, 162]). This is particularly marked during SARS-CoV, MERS-CoV and SARS-CoV-2 infection. Redox mechanisms play a role in control of coronavirus entry into mammalian cells. The ACE2 receptor and its ligand have been shown to be important for the induction of redox stress. Ang II, the ACE2 ligand, activates NADPH oxidase, increasing ROS production [162]; this is offset by ACE2, which converts Ang II to angiotensin 1–7, causing a reduction in ROS [161, 162]. Infection of cells with coronaviruses leads to reduced levels of ACE2 receptors and an increase in Ang II binding to ACE1, which causes an increase in ROS due to activation of NADPH oxidase [161, 162]. It has also been shown that S protein expression causes oxidative stress in endothelial cells, caused by activation of NOX2 [163]. Increased ROS and oxidative stress also increases the affinity of the spike protein for the human ACE2 receptor [164, 165].

Coronaviruses can induce production of mitochondrial ROS. The SARS-CoV Nsp3a protein activates the NLRP3 inflammasome, causing redistribution to the perinuclear space [166]. This activation is caused by increased mitochondrial ROS. Mitochondrial ROS regulates several pathways, which facilitate coronavirus replication in the cytoplasm. For example, it induces mitochondrial permeability transition pores, regulates endoplasmic reticulum stress and the unfolded protein response, and regulates mitophagy, which, in turn, contributes to coronavirus replication (reviewed in [161]). Most of these effects have been observed for SARS-CoV, but it seems reasonable to assume that similar effects would be observed with SARS-CoV-2.

SARS-CoV-2 can downregulate host cell antioxidant pathways, aiding replication. Specifically, it has been shown that NRF2-induced genes, such as heme oxygenase 1 (HO-1) and NAD(P)H quinone oxydoreducatse 1 (NqO1), and superoxidase dismutase (SOD), are suppressed in SARS-CoV-2-infected cells and that the NRF2 pathway inhibits SARS-CoV-2 replication, in a way quite distinct from the host inflammatory response [161, 167]. The viral Nsp14 protein interacts with the catalytic domain of the NAD-dependent deacetylase sirtuin 1 (SIRT1) and inhibits its ability to activate the NRF2/HMOX1 pathway [168]. SARS-CoV-2 also upregulates oxidative stress genes such as myeloperoxidase, calprotectin, sestrin, thioredoxin and sulfiredoxin-1 [169].

Oxidative stress induces multiple forms of DNA damage, such as single- and double-strand breaks, base and sugar oxidation products and DNA–protein crosslinks [170]. Oxidized guanine species (8-oxo-G) are usually formed by the interaction of ROS with the guanine base in nucleic acids and are taken as a measure of ROS activity. In a study to assess ROS in COVID-19 patients directly, 8-oxo-G levels were measured in cohorts of COVID-19 patients and non-infected healthy controls [171]. Increased levels of oxidized guanine were generally observed in COVID-19 patients with much higher levels in those with more severe disease. In a second study, perhaps surprisingly, similar levels of 8-oxo-G were observed in peripheral blood mononuclear cells (PBMCs) from healthy and SARS-CoV-2-infected individuals [172]. However, elevated levels of some, but not all, base excision repair proteins (for example, POLD1, MPG, ligase 1 and FEN1) and double-strand break repair proteins (for example, Chk1, RAD50 and RAD51) were reported in COVID-19 patients [172]. In addition, it was noted that more highly regulated DDR pathway protein expression was correlated with severity of disease.

Another possible source of DNA damage in SARS-CoV-2-infected cells could be the interaction of Nsp13 with DNA polymerase δ. It has been shown that the IBV protein Nsp13, which only differs from the SARS-CoV-2 orthologue in a single amino acid, binds to DNA polymerase δ p125, causing DNA replication fork stress, ATR activation, cell cycle arrest and DNA damage [138]. Inhibition of the ATR kinase by chemical inhibitors or siRNA-mediated knockdown reduced the IBV-induced ATR signalling and inhibited IBV replication [138]. It is likely that SARS-CoV-2 Nsp13 would have similar effects. Indeed, in further studies it has been shown that Nsp12 from IBV, SARS-CoV and SARS-CoV-2 interacts with the p50 regulatory subunit of pol δ [137]. This is considered to enhance the effect of Nsp13 in the induction of cell cycle arrest, as mentioned above [137].

It is also worth noting that the relatively high level of ROS could affect the viral genome as well (discussed in [173]). The effects of single amino acid substitutions in the SARS-CoV-2 S protein have been of great clinical significance during the COVID-19 pandemic. It is possible that some of these mutations could have arisen because of host cell oxidative species.

### SARS-CoV-2 and the DDR pathways

As mentioned above, probably all DNA viruses impinge on the cellular DDR [42, 44–46]. It is now becoming apparent that the same is the case for many RNA viruses [43]. SARS-CoV-2 falls into that category in that viral proteins have been shown to associate with components of the DDR pathways (reviewed in [75, 76, 174]) and the virus can affect various DDR pathways such as those based on ATR and ATM signalling [136, 175].

#### Association of SARS-CoV-2 proteins with DDR components

There have been several large-scale studies to identify the SARS-CoV-2 interactome, based on co-immunoprecipitation, yeast 2 hybrid and biotin–streptavidin based-protocols (see, for example, [158, 176–184]); in some cases, published data have been consolidated into a resource summarizing reported interactions (for example, [185]). There are appreciable variations in the binding proteins reported in the different studies but similar major groups of proteins from specific cellular pathways have been identified as significant targets for the virus. For example, host factors involved in mRNA synthesis, nuclear export, translation and stability associating with the viral N protein have been reported [158, 176, 177, 180, 181]; similarly, proteins involved in membrane trafficking have been shown to bind to Nsp7 and M as well as other viral proteins [158, 176, 177, 179]. In most reports DNA repair and damage pathways have not been considered to be a major target for interaction with SARS-CoV-2 proteins. However, many well-characterized DDR components have been identified in association with SARS-CoV-2 proteins, although it is notable that there is very limited overlap between the different studies (summarized in Table 2).

**Table 2.** DNA damage response pathway components identified as interacting with, or being in close proximity to, SARS-CoV-2 proteins by mass spectrometric analysis. A very brief description of the role of the DDR protein is given, together with appropriate references

SARS-CoV-2 protein	Associated cellular DDR protein	DDR protein function	Reference
Nsp1	RUVBL1	AAA+ATPase. DSB repair	[178]
	RUVBL2	AAA+ATPase. DSB repair	[178]
	PRIM1	DNA primase. DNA replication	[176, 177, 183]
	PRIM2	DNA primase. DNA replication	[176, 177, 183]
	POLA1	DNA polymerase alpha1. DNA replication	[158, 176–178]
	POLA2	DNA polymerase alpha2. DNA replication	[176, 177, 180]
	Timeless	Involved in replication stress and genome stability	[180]
	Ku70 (XRCC6)	DNA PK component. NHEJ double-strand break repair	[183]
	Ku80 (XRCC5)	DNA PK component. NHEJ double-strand break repair	[183]
Nsp2	Bub1B	Protein kinase. Role in mitotic checkpoint	[158]
Nsp3	Bub1B	Protein kinase. Role in mitotic checkpoint	[158]
	RCHY1	E3 ubiquitin ligase. Regulates p53 levels among others	[158]
	RINT1	RAD50 interactor 1. Regulates cell cycle and telomere length	[158]
Nsp3+Nsp8	FANCI	Fanconi anaemia component I. Role in DSB repair and repair of inter-strand crosslinks	[181]
Nsp4	POLDIP2	DNA polymerase delta interacting protein 2. Role in DNA replication	[158]
	RAD23A	A role in nucleotide excision repair of bulky lesions and oxidative DNA damage	[181]
	RINT1	RAD50 interactor 1. Regulates cell cycle and telomere length	[158]
	XPC	Nucleotide excision repair of bulky lesions and oxidative DNA damage	[181]
Nsp5	ERCC3	Nucleotide excision repair of bulky lesions and oxidative DNA damage	[176]
	FANCD2	Fanconi anaemia component D2. Role in DSB repair and repair of inter-strand crosslinks	[178]
	p53	Transcription factor, involved in cell cycle checkpoints following DNA damage	[178]
	RAD9A	Component of the 9-1-1 cell cycle checkpoint response complex	[178]
Nsp7	BRCA2	Roles in homologous recombination, G2 checkpoint control, protection of stalled replication forks	[158]
	FANCA	Fanconi anaemia component A. Role in DSB repair and repair of inter-strand crosslinks	[158]
	FANCI	Fanconi anaemia component I. Role in DSB repair and repair of inter-strand crosslinks	[158]
	Bub1	Protein kinase. Role in mitotic checkpoint	[158]
	Bub1B	Protein kinase. Role in mitotic checkpoint	[158]
	POLD1	Catalytic subunit of DNA polymerase delta. Involved in DNA replication	[178]
	POLE	Catalytic subunit of DNA polymerase epsilon. Involved in DNA replication	[158]
	Rad54B	Involved in homologous recombination	[158]
	cdc25c	Phosphatase involved in cell cycle regulation	[158]
	claspin	Adaptor protein facilitating activation of Chk1, involved in replication fork stability	[158]
	Tipin	Timeless interacting protein, involved in replication stress	[158]
	BAP1	BRCA1-associated protein 1. Role in DNA damage repair and cell cycle regulation	[158]
Nsp9	RCHY1	E3 ubiquitin ligase. Regulates p53 levels among others	[176]
	POLDIP3	DNA polymerase delta interacting protein 3. Role in DNA replication	[158]
Nsp10	POLD1	Catalytic subunit of DNA polymerase delta. Involved in DNA replication	[181]
Nsp12	FANCA	Fanconi anaemia component A. Role in DSB repair and repair of inter-strand crosslinks	[158]
Nsp14	MRE11	Exo-and endonuclease, component of the MRN complex, involved in DSB repair	[176]
	RAD50	Component of the MRN complex, involved in DSB repair	[176]
	RINT1	RAD50 interactor 1. Regulates cell cycle and telomere length	[158]
	p53	Transcription factor, involved in cell cycle checkpoints following DNA damage	[176]
	UBR5	Ubiquitin E3ligase. Regulates DNA topoisomerase II-binding protein (TopBP1) and Rnf168 in the DNA damage response	[176]
Nsp15	BLM	RecQ helicase with a role in homologous recombination	[181]
Nsp16	ERCC6L	Excision repair 6-like protein, a DNA translocase involved in the mitotic spindle	[158]
	FANCA	Fanconi anaemia component A. Role in DSB repair and repair of inter-strand crosslinks	[158]
	RINT1	RAD50 interactor 1. Regulates cell cycle and telomere length	[184]
ORF3a	SUN2	Chromosome reassembly factor	[180, 183]
	RAD23B	A role in nucleotide excision repair of bulky lesions and oxidative DNA damage	[158]
ORF3b	RAD23B	A role in nucleotide excision repair of bulky lesions and oxidative DNA damage	[158]
ORF7a	FANCI	Fanconi anaemia component I. Role in DSB repair and repair of inter-strand crosslinks	[158, 183]
	ATR	Kinase central to the DNA damage response, involved in sensing replication stress	[181]
	RAD23B	A role in nucleotide excision repair of bulky lesions and oxidative DNA damage	[158]
ORF7b	RINT1	RAD50 interactor 1. Regulates cell cycle and telomere length	[158]
ORF8	TP53RK	p53 regulating kinase	[158]
	DNMT1	DNA methyltransferase 1. Multiple functions	[177]
	POLDIP3	DNA polymerase delta interacting protein 3. Role in DNA replication	[158]
ORF9b	BRIP1	BRCA1 interacting protein1. A RecQ helicase, also known as FANCJ. Involved in homologous recombination and DNA replication stress	[178]
	hnRNPUL2	RNA-binding protein involved in homologous recombination with the MRN complex	[180]
	RAD21	Cohesin complex component. Involved in post-replicative DNA repair	[180]
	H2AX	Histone, which is central to genome stability through its role in the signalling DNA damage. Involved in the assembly of repair foci	[180]
ORF14	FANCI	Fanconi anaemia component I. Role in DSB repair and repair of inter-strand crosslinks	[158]
	RAD23B	A role in nucleotide excision repair of bulky lesions and oxidative DNA damage	[158]
	TP53RK	p53 regulating kinase	[158]
	POLDIP3	DNA polymerase delta interacting protein 3. Role in DNA replication	[158]
Envelope (E) protein	RINT1	RAD50 interactor 1. Regulates cell cycle and telomere length	[158]
	POLDIP3	DNA polymerase delta interacting protein 3. Role in DNA replication	[158]
	ZC3H18	Binds the cap-binding complex on capped RNAs	[176, 180]
Membrane (M) protein	FANCI	Fanconi anaemia component I. Role in DSB repair and repair of inter-strand crosslinks	[176, 183]
	Ligase 3	Binds to XRCC1 and is involved in base excision repair	[180]
	Timeless	Involved in replication stress and genome stability	[165]
	RINT1	RAD50 interactor 1. Regulates cell cycle and telomere length	[158]
Nucleocapsid (N) protein	hnRNPUL2	RNA-binding protein involved in homologous recombination with the MRN complex	[181]
	ERCC6L	Excision repair 6-like protein, a DNA translocase involved in the mitotic spindle	[158]
	RAD23B	A role in nucleotide excision repair of bulky lesions and oxidative DNA damage	[184]
Spike (S) protein	RINT1	RAD50 interactor 1. Regulates cell cycle and telomere length	[158]

In studies using the BioID interactome approach (which may have the disadvantage that it does not necessarily identify interacting proteins, but only those that are proximal), very large numbers of targets for each SARS-CoV-2 bait have been recorded and these include multiple DDR proteins, many associating with several SARS-CoV-2 proteins [158, 179]. For example, in the study by Samavarchi-Tehrani and colleagues, FANCI was considered to be present proximal to Nsps 1, 2, 5, 7,8, 9, 10, 13, 15 and 16, ORFs 3b, 6, 7b, 9b and 14 and the M and N proteins [179]. (Proteins identified in that study are not listed in Table 2 in order to keep it within manageable proportions.) This may be contrasted with other studies that identified FANCI interacting with Nsp3 and 7, M protein and ORF7a [158, 181, 183]. This was also the case for BRCA1, BRCA2, ERCC5, ERCC6L, XRCC5 and XRCC6, where a large number of associated SARS-CoV-2 proteins were identified for each [179]. However, this may be contrasted with other DDR proteins that appeared to be proximal to far fewer associates. For FANCA, only Nsp7 and Nsp10 were identified; for FANCM Nsp7 and 9 and ORFs 6 and 14 were proximal, and ligase III was reported to be proximal to Nsps 1, 5, 6, 10 and 15 and ORFs7b and 14 [179]. Just considering the studies summarized in Table 2, it is notable that very few of the interactors were identified in more than one study. For example, DNMT1 co-immunoprecipitates with SARS-CoV-2 ORF8 and yet was not reported as proximal to any SARS-CoV-2 protein in the studies by Laurent and colleagues and Samavarchi-Tehrani and colleagues [158, 176, 179]. Therefore, without further detailed mechanistic investigation it is difficult to know how much significance to give to these observations. For example, Laurent and colleagues and Samavarchi-Tehrani and colleagues each report interaction between Nsp7 and at least 10 DDR proteins, yet none of the other studies listed report any (Table 2). Furthermore, the BioID approach generates very large numbers of proximal targets, identified for each SARS-CoV-2 bait, sometimes exceeding a thousand [158, 179]. In that case it is not surprising that DDR proteins are included in potential binding partners.

In previous investigations it has been shown that the coronavirus infectious bronchitis virus induces cell cycle arrest by induction of an ATR-dependent DDR [138]. Additionally, the IBV Nsp13 interacts with DNA polymerase δ (POLD1), leading to DNA replication stress in IBV-infected cells [138]. Similarly, in a subsequent study it was shown that SARS-CoV-2 POLD1 probably associates with Nsp13 [137]. In the SARS-CoV-2 interactome screens summarized in Table 2, interaction of POLD1 has been reported for Nsp7 and Nsp10 but not for Nsp13 [158, 181]. However, in the BioID screen by Samavarchi-Tehrani and colleagues, POLD1 was found to be proximal to Nsps 5, 7, 8, 9, 10, 13, 15, and 16, as well as ORFs 3b, 6, 9b and 14 [179]. DNA polymerase δ is responsible for the 5′−3′ DNA polymerase and 3′−5′ exonuclease activity of polymerase δ. POLD1 has a role in several DNA repair pathways, such as mismatch repair (MMR), translesion synthesis (TLS), base excision repair (BER), nucleotide excision repair (NER) and double-strand break (DSB) repair [186].

#### Effects of SARS-CoV-2 on the cellular DDR

Obviously, further investigation is required before we can say that the reported interactions with cellular DDR components are biologically significant, either for the virus or the infected host cell. However, it is now clear that infection of mammalian cells by SARS-CoV-2 results in damage to the host cell DNA. Several studies have demonstrated this directly by comet assays and/or the presence of DNA repair foci. For example, DNA fragmentation, as measured by increased comet tail moment and the presence of micronuclei, was seen in SARS-CoV-2-infected Huh7 cells [136] and in patient cells [160]. Similarly, γH2AX foci were observed in the same cells and in SARS-CoV-2-infected lung cells from cynomolgus macaques and epithelial and endothelial lung cells from COVID-19 patients, indicative of DNA damage [136, 187, 188]. Senescence, associated with DNA damage, has also been reported following SARS-CoV-2 infection (discussed below).

As noted above, in a large proteomic screen the effects of SARS-CoV-2 on the phosphorylation of viral and host cell proteins was examined [144]. Seven viral proteins were substrates for cellular kinases at a total of 49 sites. Based on the amino acid sequences that were phosphorylated, casein kinase II (CK2), cyclin-dependent kinase (CDK), cdc2-like kinase (CLK) and protein kinase C (PKC) were highly activated. A single site probably phosphorylated by ATM or ATR was identified [144]. However, it was suggested that most of the phosphorylation sites did not play a functional role in viral replication, although the single site in Nsp9, sites in the S protein and sites towards the N-terminal region of protein N were close to proposed protein–protein interfaces [144].

On examination of cellular pathways, phosphorylation of proteins involved in double-strand break repair and single-strand DNA binding, among many others, were notably increased upon SARS-CoV-2 infection [144]. Additionally, heterochromatin and various chromosomal regions were increasingly phosphorylated. Considering more specific complexes involved in the DDR, BRCA1–HDAC1–HDAC2, BRAF53–BRCA2 and TIP5−DNMT−HDAC1 complexes were highly phosphorylated after infection. Other DDR complexes phosphorylated to a lesser extent were those comprising MGC1−DNA−PKcs−Ku, MDC1−H2AFX−TP53BP1, MDC1−p53BP1−SMC1, MDC1−MRE11−RAD50−NBS1, DNA–PK–Ku and DNA–PK–Artemis. Moreover, a complex comprising MDC1–MRN–ATM–FANCD2 was highly phosphorylated in the initial stage of SARS-CoV-2 infection but to a lesser extent later. These events are indicative of activation of double-strand break repair pathways. Overall, the activities of ~97 kinases were affected over a 24 h infection time course; the most highly activated were components of the p38 pathway, including p38ɣ (MAPK12), CK2 (CSNK2A1/2) and Ca2+/calmodulin-dependent protein kinase (CAMK2G). The activities of a large number of others was reduced, including CDK1, 2 and 5 and AKT 1 and 2 [144].

Of the 332 host proteins identified by Gordon and colleagues as interactors with viral components, 40 were significantly differentially phosphorylated following SARS-CoV-2 infection [144, 176]. Most of these had no direct involvement in the DDR; however, the phosphorylation of DNA polymerase α 2, which associates with SARS-CoV-2 Nsp1, is markedly reduced (on T^127^ and T^130^) during infection, whereas the phosphorylation of DNMT1, which binds to ORF8, is increased (on S^714^) [144]. ZC3H18 has been identified as an E protein-associated component of the DDR. Its phosphorylation on S^868^ decreases during infection, whereas phosphorylation on T^611^ initially decreases and then increases. ZC3H18 is a DNA-binding protein that promotes BRCA1 transcription, although the effects of its phosphorylation are not clear [189].

In a small pilot study, looking specifically at DDR pathways, it was observed that infection of Vero E6 cells with SARS-CoV-2 causes activation of the ATR pathway, seen as increased phosphorylation of Chk1 on S^280^ and H2AX on S^139^ [175]. In a more detailed investigation, it was observed that an ATR inhibitor, berzosertib, showed potent antiviral activity against SARS-CoV-2 in multiple cell types and blocked replication at the post-entry step [190]. Inhibition of viral replication was accompanied by a marked reduction of Chk1 phosphorylation on S^345^ [190].

Other studies have also indicated activation of DDR signalling following SARS-CoV-2 infection [136]. Over a 48 h time course, ATM and DNA-PKcs were phosphorylated on S^1981^ and S^2056^, respectively. Activation of ATM led to increased phosphorylation of KAP1 (S^824^) and H2AX (S^139^) but not Chk2 (T^68^). Furthermore, increased numbers of KAP1 S^824^, RPA S^4/8^ and γH2AX foci were seen in SARS-CoV-2-infected cells [136]. There was no significant phosphorylation of Chk1 (S^317^), but there was a marked decrease in the level of the protein detected at a later time after infection. There was also a reduction in expression of RRM2, which is normally controlled by Chk1, resulting in decreased levels of dNTPs, probably contributing to an observed reduction in S phase progression [136]. In an attempt to determine which SARS-CoV-2 proteins were responsible for Chk1 depletion, individual genes were expressed. ORF6 associates with nuclear pores, preventing the import of Chk1, leading to its accumulation in the cytoplasm and subsequent degradation by the proteasome [136]. Nsp13 also contributes to degradation of Chk1 by causing its co-localization in autophagosomes and degradation through autophagy [136]. Nsp13 had been previously reported to target TBK1 for degradation by autophagy through direct interaction and association with p62 in SARS-CoV-2-infected cells [191, 192].

Significantly, SARS-CoV-2 N protein inhibits NHEJ [136]. This appears to be through binding to dilncRNA, which inhibits the interaction with 53BP1 and its recruitment to repair foci. It has previously been reported that dilncRNA, generated at DSBs, is responsible for liquid–liquid phase separation (LLPS) of 53BP1 [193, 194]. It has been observed that 53BP1 does not readily locate to DNA repair foci in SARS-CoV-2-infected cells or following transfection of N [136]. Earlier reports had indicated that N protein condenses with RNA by LLPS and that it associates with stress granules that form through LLPS [195, 196].

Interestingly, SARS-CoV-2 has been linked to various members of the PARP [poly (ADP)-ribose polymerase] family and PARP inhibitors, such as stenoparib or olaparib, have been suggested as anti-COVID-19 agents [197–199]. However, it appears that the PARP enzymes induced by SARS-CoV-2 are MARylating PARPs (PARP7, PARP 10, PARP 12 and PARP14), which add a single ADP-ribose rather than PARPs involved in DNA repair pathways, which are generally responsible for the addition of branched or linear chains of ADP-ribose moieties [198, 200, 201]. Increased expression of the MARylating PARPs following SARS-Cov-2 infection primarily impacts on NAD metabolism, with, for example, increased levels of NAMPT (nicotinamide phosphoribosyltransferase) [198]. Thus, NAD^+^ levels were found to be reduced following increased expression of MARylating PARPs, as has been shown to be the case with PARP1 [198, 202]. The implications of these observations for any genome instability caused by SARS-CoV-2 infection are not clear at present. However, it has been demonstrated that PARP inhibitors can have anti-SARS-CoV-2 effects, although this is largely through direct inhibition of viral proteins, such as the main protease Mpro (Nsp5) [197, 203].

### SARS-CoV-2 and telomeres

In early studies, it was concluded that the severity of COVID-19 increased with age, with older patients having higher mortality (see, for example, [204–206]). Therefore, molecular pathways underlying aging, such as telomere shortening, were thought to be important in determining the severity of the disease. Indeed, it has been reported that patients with a larger proportion of short telomeres developed more severe COVID-19 symptoms [207, 208]. However, this is not a view that is universally held and other reports have indicated that decreased telomere length is not necessarily linked to increased severity of COVID-19 (for example, [209, 210]).

The production of lymphocytes is closely linked to telomere length. Shorter telomeres due to aging can induce slow proliferation of lymphocytes. To achieve a requisite immune response to SARS-CoV-2 infection, generation and maintenance of T-cells is essential. Marked reduction of the T-cell count, lymphopenia, has been found to be a characteristic of severe COVID-19. In older patients infected with SARS-CoV-2, PBMC telomeres have been observed to be relatively short and this was linked to low lymphocyte counts. T-cell proliferative capacity is telomere length-dependent, and telomeres shorten with age. These observations may account for the vulnerability of older patients to COVID-19 [211]. In other studies, it has been shown that short blood leukocyte telomere lengths, as a marker of age, can contribute to the development of post-COVID-19 symptoms in the lungs of some patients [212].

Interestingly, expression of the ACE2 receptor increases with telomere shortening in mice and in cultured human cells [213]. Regulation is at the transcriptional level with the ACE2 promoter being DDR-dependent. Thus, ATM inhibition or inhibition of the telomeric DDR leads to increased ACE2 level. It has been concluded that during aging there is increased telomere shortening, increased DDR activity and increased ACE2 expression, resulting in more severe levels of SARS-CoV-2 infection [213]. Importantly, ATM and ATR activities are required for localization of telomerase to telomeres and telomere elongation [214]. It might be supposed in the case of prolonged infection, SARS-CoV-2 could cause alteration in telomere length by affecting PIKKs.

### Senescence induced by SARS-CoV-2

Simplistically, two major forms of senescence can be delineated. Replicative senescence is associated with the shortening of telomeres with increasing age, whereas stress-induced senescence is a response to a wide variety of factors, including DNA damage and infection by viruses such as SARS-Cov-2, irrespective of telomere length [215–217]. It has now been shown that SARS-CoV-2 can induce virus-induced-senescence (VIS) by a number of different mechanisms (reviewed in [217, 218]). Many virus types, including single- and double-stranded DNA and RNA viruses can cause VIS, which is generally very similar to oncogene-induced senescence (reviewed in [217]). Infection with SARS-CoV-2 has been shown to be responsible for senescence-associated secretory phenotype (SASP), which involves secretion of pro-inflammatory cytokines such as IL-1β, IL-6 and Il-8 and the presence of γH2AX foci, which is probably due to the presence of ROS [219]. Reduction in ROS in *in vitro* models reduced DNA damage and senescence-associated β-galactosidase activity. Shedding of mitochondrial outer membranes in response to stress caused by infection could also contribute to DNA damage caused by the virus [220–222]. VIS is also marked by increased expression of the cell cycle inhibitors p16 and p21 and the inhibition of expression of cell cycle promoters, such as E2F targets and SASP [217]. (SASP is characterized by secretion of extracellular modifying factors, such as pro-inflammatory factors, ECM-degrading proteins and pro-coagulatory factors, amongst others [217]).

## Long COVID and DNA damage

Long COVID [also referred to as ‘post-COVID condition’, ‘post-COVID syndrome’ and ‘post-acute sequelae of COVID-19’ (PASC)] is a prolonged, often severe condition with diverse symptoms occurring after SARS-CoV-2 infection (reviewed in [7, 223–227]). Symptoms include fatigue, anosmia and dysgeusia, persistent respiratory problems, such as shortness of breath, periodic cognitive difficulties, cardiovascular difficulties, such as chest pain and arrhythmia, gastrointestinal symptoms, rheumatic and dermatological problems, and neurological and psychiatric symptoms (reviewed, for example, in [224]). Because of the multiple, diverse symptoms associated with long COVID, explanations of its causes have been many and varied. For example, it has been suggested that it is due to residual virus, immune dysregulation, possibly due to reactivation of a latent herpesvirus, more general effects of SARS-CoV-2 on the host microbiota, autoimmunity and/or residual dysfunctional signalling (reviewed in [7]). Of particular interest from the point of view of long-term damage to the DNA and the induction of genome instability is the possibility that there is residual or perhaps latent virus remaining in the infected patient, ostensibly after recovery. It is now clear that components of SARS-CoV-2 are relatively common after the disappearance of symptoms. Two forms of persistence can be recognized [223, 227]. Firstly, the presence of persistent infectious SARS-CoV-2, capable of replicating. It has been suggested that persistent viral reservoirs could be present in patients, even though they test negative by PCR [228]. SARS-CoV-2 RNA was shown to be present at multiple sites within the bodies of a cohort of patients who had died from COVID-19 [229]. Virus could be isolated from approximately half of the tissues tested; in one case over 7 months since diagnosis [229]. Other studies have shown SARS-CoV-2 in sites such as the intestine, respiratory tract and lymph nodes [230–232]. The second form of persistence would be the retention of viral RNA and/or protein at specific sites. For example, SARS-CoV-2 N protein was detected in intestinal epithelium 4 months after COVID-19 diagnosis in a third of patients in one study. Viral RNA was detected in half of those who were positive for N protein, and all tested negative by PCR for the virus [233]. SARS-CoV-2 RNA has been detected in the kidney, respiratory tract and blood 2 months after infection [234–238]. In a further study, SARS-CoV-2 N protein was also observed at several sites up to 6 months after testing negative by PCR in a small cohort of patients [239].

Further evidence suggests that reverse-transcribed SARS-CoV-2 RNA can be integrated into host cell DNA with subsequent transcription of the integrated sequences [240]. These observations were made primarily on cells in culture, but it was also noted that in some patient-derived samples viral sequences were transcribed from integrated DNA copies of viral sequences, although these small fragments were not capable of producing infectious virus [240]. It should, however, be noted that these suggestions are controversial and have been contradicted in other reports [241, 242].

It is tempting to compare the effects of SARS-CoV-2 infection with those of HCV, another positive-sense, single-stranded RNA virus. HCV establishes chronic or persistent low-level infection in the liver, and this is a major contributory factor (with HBV) to hepatitis and hepatocellular carcinoma (HCC). Chronic HCV infection causes oxidative stress and high levels of ROS, as well as stimulating the production of NO via activation of inducible NO synthase (iNOS) [243–250]. The effects of these high levels of NO and ROS probably lead to the accumulation of DNA damage and the genetic abnormalities observed in HCV-infected cells; this can drive the progression of HCV-associated malignancies [246, 247, 249]. As well as inducing DNA damage, HCV proteins can directly impinge on the cellular DDR, generally reducing its effectiveness (reviewed in [43, 251]).

Chronic infection by HCV can initiate a non-specific immune-mediated inflammation, seen as hepatitis, which is linked to oxidative stress. As discussed above, SARS-CoV-2 infection is closely associated with inflammation and the generation of ROS. If the virus were to persist in a comparable fashion to HCV it is possible that it could also have serious deleterious consequences for the infected tissue and possibly cause DNA damage and genomic instability. Time will tell if this is the case.

## Conclusions

It is less than 4 years since the initial outbreak of infection in Wuhan by a previously unknown virus. Yet our knowledge of many facets of SARS-CoV-2’s life cycle, replication and effects on the infected host cell is considerable, although there are aspects of SARS-CoV-2 biology that have not, up to now, been examined in detail. One of these is the relationship between the virus and the cellular DDR, although it is now becoming clear that some of these pathways are a target for the virus, although perhaps in a minor way.

Importantly, as well as impinging on DDR pathways, SARS-CoV-2 causes damage to cellular DNA, generally through the generation of ROS, which can cause double- and single-strand breaks, intra- and inter-strand crosslinks, and non-bulky and bulky base modifications. ROS are generated at several points during viral infection, generally through activation of multiple inflammatory pathways, as outlined above. These effects are amplified by the ability of the virus to downregulate host cell antioxidant pathways, aiding replication and possible upregulation of pro-oxidant genes.

Although there have been several large-scale studies examining the interactome for SARS-CoV-2 proteins, the results have tended to be somewhat contradictory (see above). However, components of different DDR pathways have been identified as binding partners for viral proteins, such as the association of POLD1 with Nsp7 and Nsp10 [158, 181], the association of MRE11 and RAD50 with Nsp14 [161], and the association of POLA1 and POLA2 with Nsp1 [158, 176–178, 180, 181]. Most of the reported interactions are summarized in Table 2. Whether they are all significant, or even occur *in vivo*, is open to question, although it seems reasonable to assume that these data establish that DDR pathways are targets for SARS-CoV-2. As well as associating with DDR proteins, it has been confirmed that SARS-CoV-2 proteins affect DDR pathways. For example, SARS-CoV-2 infection results in phosphorylation of ATM and DNA-PKcs, an increase in DNA repair foci containing phosphorylated ATM substrates and degradation of Chk1 [136]. It seems that there is a requirement for certain DDR pathways for optimal viral replication as addition of the ATR inhibitor, berzosertib, has potent anti-viral activity, inhibiting Chk1 phosphorylation [190].

The effects of SARS-CoV-2 on the host genome and on the cellular DDR are comparable to those seen with other RNA and DNA viruses. Infection with the RNA viruses HIV-1, HTLV-1 and HCV generates ROS, which can cause DNA damage (reviewed in [43]). In the case of HCV (and HBV), this has been linked to hepatocellular carcinoma. Similarly, ROS production is also a consequence of DNA virus infection, such as is observed with, among others, HBV, EBV and HPV [42, 252]. Although our knowledge of the effects of SARS-CoV-2 on the DDR is not extensive, again they appear to be similar to those reported for other viruses. Thus, there is an activation of ATM, and DNA-PK signalling, as is generally observed, and a generation of γH2AX repair foci [42, 46, 136]. A point of some concern may be whether SARS-CoV-2 virus or viral proteins and/or RNA persist in patients suffering from long COVID and whether these could cause genome instability over the longer term. If this were the case, a rise in cancer cases might be seen in future.

## References

[1] Almas T, Malik J, Alsubai AK, Jawad Zaidi SM, Iqbal R (2022). Post-acute COVID-19 syndrome and its prolonged effects: an updated systematic review. Ann Med Surg.

[2] Du M, Ma Y, Deng J, Liu M, Liu J (2022). Comparison of long COVID-19 caused by different SARS-CoV-2 strains: a systematic review and meta-analysis. Int J Environ Res Public Health.

[3] Montani D, Savale L, Noel N, Meyrignac O, Colle R (2022). Post-acute COVID-19 syndrome. Eur Respir Rev.

[4] O’Mahoney LL, Routen A, Gillies C, Ekezie W, Welford A (2023). The prevalence and long-term health effects of long Covid among hospitalised and non-hospitalised populations: a systematic review and meta-analysis. EClinicalMedicine.

[5] O’Mahoney L, Khunti K (2022). Long covid: risk factors, outcomes, and future directions for research. BMJ Med.

[6] Deer RR, Rock MA, Vasilevsky N, Carmody L, Rando H (2021). Characterizing long COVID: deep phenotype of a complex condition. EBioMedicine.

[7] Davis HE, McCorkell L, Vogel JM, Topol EJ (2023). Long COVID: major findings, mechanisms and recommendations. Nat Rev Microbiol.

[8] Zhou P, Yang X-L, Wang X-G, Hu B, Zhang L (2020). A pneumonia outbreak associated with a new coronavirus of probable bat origin. Nature.

[9] Zhu N, Zhang D, Wang W, Li X, Yang B (2020). A novel coronavirus from patients with pneumonia in China, 2019. N Engl J Med.

[10] Lu R, Zhao X, Li J, Niu P, Yang B (2020). Genomic characterisation and epidemiology of 2019 novel coronavirus: implications for virus origins and receptor binding. Lancet.

[11] Kim J-M, Chung Y-S, Jo HJ, Lee N-J, Kim MS (2020). Identification of coronavirus isolated from a patient in Korea with COVID-19. Osong Public Health Res Perspect.

[12] V’kovski P, Kratzel A, Steiner S, Stalder H, Thiel V (2021). Coronavirus biology and replication: implications for SARS-CoV-2. Nat Rev Microbiol.

[13] Hartenian E, Nandakumar D, Lari A, Ly M, Tucker JM (2020). The molecular virology of coronaviruses. J Biol Chem.

[14] Jahirul Islam M, Nawal Islam N, Siddik Alom M, Kabir M, Halim MA (2023). A review on structural, non-structural, and accessory proteins of SARS-CoV-2: highlighting drug target sites. Immunobiology.

[15] Kaur N, Singh R, Dar Z, Bijarnia RK, Dhingra N (2021). Genetic comparison among various coronavirus strains for the identification of potential vaccine targets of SARS-CoV2. Infect Genet Evol.

[16] Yang H, Rao Z (2021). Structural biology of SARS-CoV-2 and implications for therapeutic development. Nat Rev Microbiol.

[17] Wu F, Zhao S, Yu B, Chen Y-M, Wang W (2020). A new coronavirus associated with human respiratory disease in China. Nature.

[18] Fan K, Wei P, Feng Q, Chen S, Huang C (2004). Biosynthesis, purification, and substrate specificity of severe acute respiratory syndrome coronavirus 3C-like proteinase. J Biol Chem.

[19] Harcourt BH, Jukneliene D, Kanjanahaluethai A, Bechill J, Severson KM (2004). Identification of severe acute respiratory syndrome coronavirus replicase products and characterization of papain-like protease activity. J Virol.

[20] Jin Z, Du X, Xu Y, Deng Y, Liu M (2020). Structure of Mpro from SARS-CoV-2 and discovery of its inhibitors. Nature.

[21] Klemm T, Ebert G, Calleja DJ, Allison CC, Richardson LW (2020). Mechanism and inhibition of the papain-like protease, PLpro, of SARS-CoV-2. EMBO J.

[22] Blackford AN, Jackson SP (2017). ATM, ATR, and DNA-PK: the trinity at the heart of the DNA damage response. Mol Cell.

[23] Ciccia A, Elledge SJ (2010). The DNA damage response: making it safe to play with knives. Mol Cell.

[24] Davis AJ, Chen DJ (2013). DNA double strand break repair via non-homologous end-joining. Transl Cancer Res.

[25] Chang HHY, Pannunzio NR, Adachi N, Lieber MR (2017). Non-homologous DNA end joining and alternative pathways to double-strand break repair. Nat Rev Mol Cell Biol.

[26] Spies J, Polasek-Sedlackova H, Lukas J, Somyajit K (2021). Homologous recombination as a fundamental genome surveillance mechanism during DNA replication. Genes.

[27] Wright WD, Shah SS, Heyer WD (2018). Homologous recombination and the repair of DNA double-strand breaks. J Biol Chem.

[28] Krejci L, Altmannova V, Spirek M, Zhao X (2012). Homologous recombination and its regulation. Nucleic Acids Res.

[29] Li Z, Pearlman AH, Hsieh P (2016). DNA mismatch repair and the DNA damage response. DNA Repair.

[30] Li G-M (2008). Mechanisms and functions of DNA mismatch repair. Cell Res.

[31] Ijsselsteijn R, Jansen JG, de Wind N (2020). DNA mismatch repair-dependent DNA damage responses and cancer. DNA Repair.

[32] Fishel R (2015). Mismatch repair. J Biol Chem.

[33] Hegde ML, Hazra TK, Mitra S (2008). Early steps in the DNA base excision/single-strand interruption repair pathway in mammalian cells. Cell Res.

[34] Dianov GL, Hübscher U (2013). Mammalian base excision repair: the forgotten archangel. Nucleic Acids Res.

[35] Spivak G (2015). Nucleotide excision repair in humans. DNA Repair.

[36] Reardon JT, Sancar A (2005). Nucleotide excision repair. Prog Nucleic Acid Res Mol Biol.

[37] Ceccaldi R, Sarangi P, D’Andrea AD (2016). The Fanconi anaemia pathway: new players and new functions. Nat Rev Mol Cell Biol.

[38] D’Andrea AD, Grompe M (2003). The Fanconi anaemia/BRCA pathway. Nat Rev Cancer.

[39] Taylor AMR, Rothblum-Oviatt C, Ellis NA, Hickson ID, Meyer S (2019). Chromosome instability syndromes. Nat Rev Dis Primers.

[40] Kaina B, Christmann M, Naumann S, Roos WP (2007). MGMT: key node in the battle against genotoxicity, carcinogenicity and apoptosis induced by alkylating agents. DNA Repair.

[41] Gutierrez R, O’Connor TR (2021). DNA direct reversal repair and alkylating agent drug resistance. Cancer Drug Resist.

[42] Hollingworth R, Grand RJ (2015). Modulation of DNA damage and repair pathways by human tumour viruses. Viruses.

[43] Ryan EL, Hollingworth R, Grand RJ (2016). Activation of the DNA damage response by RNA viruses. Biomolecules.

[44] Turnell AS, Grand RJ (2012). DNA viruses and the cellular DNA-damage response. J Gen Virol.

[45] Pancholi NJ, Price AM, Weitzman MD (2017). Take your PIKK: tumour viruses and DNA damage response pathways. Philos Trans R Soc Lond B Biol Sci.

[46] Weitzman MD, Fradet-Turcotte A (2018). Virus DNA replication and the host DNA damage response. Annu Rev Virol.

[47] Luftig MA (2014). Viruses and the DNA damage response: activation and antagonism. Annu Rev Virol.

[48] Weitzman MD, Lilley CE, Chaurushiya MS (2010). Genomes in conflict: maintaining genome integrity during virus infection. Annu Rev Microbiol.

[49] Stracker TH, Carson CT, Weitzman MD (2002). Adenovirus oncoproteins inactivate the Mre11-Rad50-NBS1 DNA repair complex. Nature.

[50] Orazio NI, Naeger CM, Karlseder J, Weitzman MD (2011). The adenovirus E1b55K/E4orf6 complex induces degradation of the bloom helicase during infection. J Virol.

[51] Chaurushiya MS, Lilley CE, Aslanian A, Meisenhelder J, Scott DC (2012). Viral E3 ubiquitin ligase-mediated degradation of a cellular E3: viral mimicry of a cellular phosphorylation mark targets the RNF8 FHA domain. Mol Cell.

[52] Parkinson J, Lees-Miller SP, Everett RD (1999). Herpes simplex virus type 1 immediate-early protein vmw110 induces the proteasome-dependent degradation of the catalytic subunit of DNA-dependent protein kinase. J Virol.

[53] Xiaofei E, Kowalik TF (2014). The DNA damage response induced by infection with human cytomegalovirus and other viruses. Viruses.

[54] Hagemeier SR, Barlow EA, Meng Q, Kenney SC (2012). The cellular ataxia telangiectasia-mutated kinase promotes epstein-barr virus lytic reactivation in response to multiple different types of lytic reactivation-inducing stimuli. J Virol.

[55] Hollingworth R, Horniblow RD, Forrest C, Stewart GS, Grand RJ (2017). Localization of double-strand break repair proteins to viral replication compartments following lytic reactivation of Kaposi’s sarcoma-associated herpesvirus. J Virol.

[56] Lilley CE, Carson CT, Muotri AR, Gage FH, Weitzman MD (2005). DNA repair proteins affect the lifecycle of herpes simplex virus 1. Proc Natl Acad Sci U S A.

[57] Li R, Zhu J, Xie Z, Liao G, Liu J (2011). Conserved herpesvirus kinases target the DNA damage response pathway and TIP60 histone acetyltransferase to promote virus replication. Cell Host Microbe.

[58] Shi Y, Dodson GE, Shaikh S, Rundell K, Tibbetts RS (2005). Ataxia-telangiectasia-mutated (ATM) is a T-antigen kinase that controls SV40 viral replication in vivo. J Biol Chem.

[59] Dahl J, You J, Benjamin TL (2005). Induction and utilization of an ATM signaling pathway by polyomavirus. J Virol.

[60] Hollingworth R, Skalka GL, Stewart GS, Hislop AD, Blackbourn DJ (2015). Activation of DNA damage response pathways during lytic replication of KSHV. Viruses.

[61] Blackford AN, Bruton RK, Dirlik O, Stewart GS, Taylor AMR (2008). A role for E1B-AP5 in ATR signaling pathways during adenovirus infection. J Virol.

[62] Daikoku T, Kudoh A, Sugaya Y, Iwahori S, Shirata N (2006). Postreplicative mismatch repair factors are recruited to Epstein-Barr virus replication compartments. J Biol Chem.

[63] Gillespie KA, Mehta KP, Laimins LA, Moody CA (2012). Human papillomaviruses recruit cellular DNA repair and homologous recombination factors to viral replication centers. J Virol.

[64] Hau PM, Deng W, Jia L, Yang J, Tsurumi T (2015). Role of ATM in the formation of the replication compartment during lytic replication of Epstein-Barr virus in nasopharyngeal epithelial cells. J Virol.

[65] Tsang SH, Wang X, Li J, Buck CB, You J (2014). Host DNA damage response factors localize to merkel cell polyomavirus DNA replication sites to support efficient viral DNA replication. J Virol.

[66] Denison MR (2008). Seeking membranes: positive-strand RNA virus replication complexes. PLoS Biol.

[67] Zawilska JB, Lagodzinski A, Berezinska M (2021). COVID-19: from the structure and replication cycle of SARS-CoV-2 to its disease symptoms and treatment. J Physiol Pharmacol.

[68] Howley PM, Knipe DM, Whelan S, Freed EO, Cohen JL (2022).

[69] Clavarino G, Cláudio N, Couderc T, Dalet A, Judith D (2012). Induction of GADD34 is necessary for dsRNA-dependent interferon-β production and participates in the control of Chikungunya virus infection. PLoS Pathog.

[70] Nargi-Aizenman JL, Simbulan-Rosenthal CM, Kelly TA, Smulson ME, Griffin DE (2002). Rapid activation of poly(ADP-ribose) polymerase contributes to Sindbis virus and staurosporine-induced apoptotic cell death. Virology.

[71] Tachiwana H, Shimura M, Nakai-Murakami C, Tokunaga K, Takizawa Y (2006). HIV-1 Vpr induces DNA double-strand breaks. Cancer Res.

[72] Nakai-Murakami C, Shimura M, Kinomoto M, Takizawa Y, Tokunaga K (2007). HIV-1 Vpr induces ATM-dependent cellular signal with enhanced homologous recombination. Oncogene.

[73] Li N, Parrish M, Chan TK, Yin L, Rai P (2015). Influenza infection induces host DNA damage and dynamic DNA damage responses during tissue regeneration. Cell Mol Life Sci.

[74] Vijaya Lakshmi AN, Ramana MV, Vijayashree B, Ahuja YR, Sharma G (1999). Detection of influenza virus induced DNA damage by comet assay. Mutat Res.

[75] Pánico P, Ostrosky-Wegman P, Salazar AM (2022). The potential role of COVID-19 in the induction of DNA damage. Mutat Res Rev Mutat Res.

[76] Papanikolaou C, Rapti V, Stellas D, Stefanou DT, Syrigos K (2022). Delineating the SARS-CoV-2 induced interplay between the host immune system and the DNA damage response network. Vaccines.

[77] Li W, Moore MJ, Vasilieva N, Sui J, Wong SK (2003). Angiotensin-converting enzyme 2 is a functional receptor for the SARS coronavirus. Nature.

[78] Letko M, Marzi A, Munster V (2020). Functional assessment of cell entry and receptor usage for SARS-CoV-2 and other lineage B betacoronaviruses. Nat Microbiol.

[79] Hoffmann M, Kleine-Weber H, Schroeder S, Krüger N, Herrler T (2020). SARS-CoV-2 cell entry depends on ACE2 and TMPRSS2 and is blocked by a clinically proven protease inhibitor. Cell.

[80] Lan J, Ge J, Yu J, Shan S, Zhou H (2020). Structure of the SARS-CoV-2 spike receptor-binding domain bound to the ACE2 receptor. Nature.

[81] Walls AC, Park Y-J, Tortorici MA, Wall A, McGuire AT (2020). Structure, function, and antigenicity of the SARS-CoV-2 spike glycoprotein. Cell.

[82] Hoffmann M, Kleine-Weber H, Pöhlmann S (2020). A multibasic cleavage site in the spike protein of SARS-CoV-2 is essential for infection of human lung cells. Mol Cell.

[83] Sawicki SG, Sawicki DL (2005). Coronavirus transcription: a perspective. Curr Top Microbiol Immunol.

[84] Kim D, Lee J-Y, Yang J-S, Kim JW, Kim VN (2020). The architecture of SARS-CoV-2 transcriptome. Cell.

[85] Sola I, Almazán F, Zúñiga S, Enjuanes L (2015). Continuous and discontinuous RNA synthesis in coronaviruses. Annu Rev Virol.

[86] Finkel Y, Mizrahi O, Nachshon A, Weingarten-Gabbay S, Morgenstern D (2021). The coding capacity of SARS-CoV-2. Nature.

[87] Lei J, Kusov Y, Hilgenfeld R (2018). Nsp3 of coronaviruses: structures and functions of a large multi-domain protein. Antiviral Res.

[88] Zhang L, Lin D, Sun X, Curth U, Drosten C (2020). Crystal structure of SARS-CoV-2 main protease provides a basis for design of improved α-ketoamide inhibitors. Science.

[89] Scott BM, Lacasse V, Blom DG, Tonner PD, Blom NS (2022). Predicted coronavirus Nsp5 protease cleavage sites in the human proteome. BMC Genom Data.

[90] Thoms M, Buschauer R, Ameismeier M, Koepke L, Denk T (2020). Structural basis for translational shutdown and immune evasion by the Nsp1 protein of SARS-CoV-2. Science.

[91] Schubert K, Karousis ED, Jomaa A, Scaiola A, Echeverria B (2020). SARS-CoV-2 Nsp1 binds the ribosomal mRNA channel to inhibit translation. Nat Struct Mol Biol.

[92] Kamitani W, Huang C, Narayanan K, Lokugamage KG, Makino S (2009). A two-pronged strategy to suppress host protein synthesis by SARS coronavirus Nsp1 protein. Nat Struct Mol Biol.

[93] Kakavandi S, Zare I, VaezJalali M, Dadashi M, Azarian M (2023). Structural and non-structural proteins in SARS-CoV-2: potential aspects to COVID-19 treatment or prevention of progression of related diseases. Cell Commun Signal.

[94] Arya R, Kumari S, Pandey B, Mistry H, Bihani SC (2021). Structural insights into SARS-CoV-2 proteins. J Mol Biol.

[95] Gao Y, Yan L, Huang Y, Liu F, Zhao Y (2020). Structure of the RNA-dependent RNA polymerase from COVID-19 virus. Science.

[96] Kirchdoerfer RN, Ward AB (2019). Structure of the SARS-CoV nsp12 polymerase bound to nsp7 and nsp8 co-factors. Nat Commun.

[97] Konkolova E, Klima M, Nencka R, Boura E (2020). Structural analysis of the putative SARS-CoV-2 primase complex. J Struct Biol.

[98] Ma Y, Wu L, Shaw N, Gao Y, Wang J (2015). Structural basis and functional analysis of the SARS coronavirus nsp14-nsp10 complex. Proc Natl Acad Sci U S A.

[99] Baddock HT, Brolih S, Yosaatmadja Y, Ratnaweera M, Bielinski M (2022). Characterization of the SARS-CoV-2 ExoN (nsp14ExoN-nsp10) complex: implications for its role in viral genome stability and inhibitor identification. Nucleic Acids Res.

[100] Ogando NS, Zevenhoven-Dobbe JC, van der Meer Y, Bredenbeek PJ, Posthuma CC (2020). The enzymatic activity of the nsp14 exoribonuclease is critical for replication of MERS-CoV and SARS-CoV-2. J Virol.

[101] Chen J, Malone B, Llewellyn E, Grasso M, Shelton PMM (2020). Structural basis for helicase-polymerase coupling in the SARS-CoV-2 replication-transcription complex. Cell.

[102] Newman JA, Douangamath A, Yadzani S, Yosaatmadja Y, Aimon A (2021). Structure, mechanism and crystallographic fragment screening of the SARS-CoV-2 NSP13 helicase. Nat Commun.

[103] Huang T, Snell KC, Kalia N, Gardezi S, Guo L (2023). Kinetic analysis of RNA cleavage by coronavirus Nsp15 endonuclease: evidence for acid-base catalysis and substrate-dependent metal ion activation. J Biol Chem.

[104] Frazier MN, Dillard LB, Krahn JM, Perera L, Williams JG (2021). Characterization of SARS2 Nsp15 nuclease activity reveals it’s mad about U. Nucleic Acids Res.

[105] Nencka R, Silhan J, Klima M, Otava T, Kocek H (2022). Coronaviral RNA-methyltransferases: function, structure and inhibition. Nucleic Acids Res.

[106] Krafcikova P, Silhan J, Nencka R, Boura E (2020). Structural analysis of the SARS-CoV-2 methyltransferase complex involved in RNA cap creation bound to sinefungin. Nat Commun.

[107] Lu S, Ye Q, Singh D, Cao Y, Diedrich JK (2021). The SARS-CoV-2 nucleocapsid phosphoprotein forms mutually exclusive condensates with RNA and the membrane-associated M protein. Nat Commun.

[108] Zheng Y, Zhuang M-W, Han L, Zhang J, Nan M-L (2020). Severe acute respiratory syndrome coronavirus 2 (SARS-CoV-2) membrane (M) protein inhibits type I and III interferon production by targeting RIG-I/MDA-5 signaling. Signal Transduct Target Ther.

[109] Lei X, Dong X, Ma R, Wang W, Xiao X (2020). Activation and evasion of type I interferon responses by SARS-CoV-2. Nat Commun.

[110] Zhang Z, Nomura N, Muramoto Y, Ekimoto T, Uemura T (2022). Structure of SARS-CoV-2 membrane protein essential for virus assembly. Nat Commun.

[111] Marques-Pereira C, Pires MN, Gouveia RP, Pereira NN, Caniceiro AB (2022). SARS-CoV-2 membrane protein: from genomic data to structural new insights. Int J Mol Sci.

[112] Bai Z, Cao Y, Liu W, Li J (2021). The SARS-CoV-2 nucleocapsid protein and its role in viral structure, biological functions, and a potential target for drug or vaccine mitigation. Viruses.

[113] Savastano A, Ibáñez de Opakua A, Rankovic M, Zweckstetter M (2020). Nucleocapsid protein of SARS-CoV-2 phase separates into RNA-rich polymerase-containing condensates. Nat Commun.

[114] Gao T, Gao Y, Liu X, Nie Z, Sun H (2021). Identification and functional analysis of the SARS-COV-2 nucleocapsid protein. BMC Microbiol.

[115] Wu W, Cheng Y, Zhou H, Sun C, Zhang S (2023). The SARS-CoV-2 nucleocapsid protein: its role in the viral life cycle, structure and functions, and use as a potential target in the development of vaccines and diagnostics. Virol J.

[116] Perdikari TM, Murthy AC, Ryan VH, Watters S, Naik MT (2020). SARS‐CoV‐2 nucleocapsid protein phase‐separates with RNA and with human hnRNPs. The EMBO Journal.

[117] Zhu C, He G, Yin Q, Zeng L, Ye X (2021). Molecular biology of the SARs-CoV-2 spike protein: a review of current knowledge. J Med Virol.

[118] Wrapp D, Wang N, Corbett KS, Goldsmith JA, Hsieh C-L (2020). Cryo-EM structure of the 2019-nCoV spike in the prefusion conformation. Science.

[119] Yan R, Zhang Y, Li Y, Xia L, Guo Y (2020). Structural basis for the recognition of SARS-CoV-2 by full-length human ACE2. Science.

[120] Cao Y, Yang R, Lee I, Zhang W, Sun J (2021). Characterization of the SARS-CoV-2 E protein: sequence, structure, viroporin, and inhibitors. Protein Sci.

[121] Zhou S, Lv P, Li M, Chen Z, Xin H (2023). SARS-CoV-2 E protein: pathogenesis and potential therapeutic development. Biomed Pharmacother.

[122] Redondo N, Zaldívar-López S, Garrido JJ, Montoya M (2021). SARS-CoV-2 accessory proteins in viral pathogenesis: knowns and unknowns. Front Immunol.

[123] Hurtado-Tamayo J, Requena-Platek R, Enjuanes L, Bello-Perez M, Sola I (2023). Contribution to pathogenesis of accessory proteins of deadly human coronaviruses. Front Cell Infect Microbiol.

[124] Shang J, Han N, Chen Z, Peng Y, Li L (2021). Compositional diversity and evolutionary pattern of coronavirus accessory proteins. Brief Bioinform.

[125] Xia H, Cao Z, Xie X, Zhang X, Chen JY-C (2020). Evasion of type I interferon by SARS-CoV-2. Cell Rep.

[126] Rashid F, Xie Z, Suleman M, Shah A, Khan S (2022). Roles and functions of SARS-CoV-2 proteins in host immune evasion. Front Immunol.

[127] Zandi M, Shafaati M, Kalantar-Neyestanaki D, Pourghadamyari H, Fani M (2022). The role of SARS-CoV-2 accessory proteins in immune evasion. Biomed Pharmacother.

[128] Zhou BB, Elledge SJ (2000). The DNA damage response: putting checkpoints in perspective. Nature.

[129] Lukas J, Lukas C, Bartek J (2004). Mammalian cell cycle checkpoints: signalling pathways and their organization in space and time. DNA Repair.

[130] Nascimento R, Costa H, Parkhouse RME (2012). Virus manipulation of cell cycle. Protoplasma.

[131] Berk AJ (2005). Recent lessons in gene expression, cell cycle control, and cell biology from adenovirus. Oncogene.

[132] Ben-Israel H, Kleinberger T (2002). Adenovirus and cell cycle control. Front Biosci.

[133] Chiu Y-F, Sugden AU, Sugden B (2013). Epstein-Barr viral productive amplification reprograms nuclear architecture, DNA replication, and histone deposition. Cell Host Microbe.

[134] Hollingworth R, Stewart GS, Grand RJ (2020). Productive herpesvirus lytic replication in primary effusion lymphoma cells requires S-phase entry. J Gen Virol.

[135] Baer A, Austin D, Narayanan A, Popova T, Kainulainen M (2012). Induction of DNA damage signaling upon Rift Valley fever virus infection results in cell cycle arrest and increased viral replication. J Biol Chem.

[136] Gioia U, Tavella S, Martínez-Orellana P, Cicio G, Colliva A (2023). SARS-CoV-2 infection induces DNA damage, through CHK1 degradation and impaired 53BP1 recruitment, and cellular senescence. Nat Cell Biol.

[137] Quan L, Sun X, Xu L, Chen RA, Liu DX (2023). Coronavirus RNA-dependent RNA polymerase interacts with the p50 regulatory subunit of host DNA polymerase delta and plays a synergistic role with RNA helicase in the induction of DNA damage response and cell cycle arrest in the S phase. Emerg Microbes Infect.

[138] Xu LH, Huang M, Fang SG, Liu DX (2011). Coronavirus infection induces DNA replication stress partly through interaction of its nonstructural protein 13 with the p125 subunit of DNA polymerase δ. J Biol Chem.

[139] Wang W, Chen J, Hu D, Pan P, Liang L (2022). SARS-CoV-2 N protein induces acute kidney injury via Smad3-dependent G1 cell cycle arrest mechanism. Adv Sci.

[140] Wu W, Wang W, Liang L, Chen J, Wei B (2023). Treatment with quercetin inhibits SARS-CoV-2 N protein-induced acute kidney injury by blocking Smad3-dependent G1 cell-cycle arrest. Mol Ther.

[141] Gao T, Gao Y, Liu X, Nie Z, Sun H (2021). Identification and functional analysis of the SARS-COV-2 nucleocapsid protein. BMC Microbiol.

[142] Surjit M, Liu B, Chow VTK, Lal SK (2006). The nucleocapsid protein of severe acute respiratory syndrome-coronavirus inhibits the activity of cyclin-cyclin-dependent kinase complex and blocks S phase progression in mammalian cells. J Biol Chem.

[143] Su M, Chen Y, Qi S, Shi D, Feng L (2020). A mini-review on cell cycle regulation of coronavirus infection. Front Vet Sci.

[144] Bouhaddou M, Memon D, Meyer B, White KM, Rezelj VV (2020). The global phosphorylation landscape of SARS-CoV-2 infection. Cell.

[145] Lee J, Hong F, Kwon S, Kim SS, Kim DO (2002). Activation of p38 MAPK induces cell cycle arrest via inhibition of Raf/ERK pathway during muscle differentiation. Biochem Biophys Res Commun.

[146] Parker LL, Piwnica-Worms H (1992). Inactivation of the p34cdc2-cyclin B complex by the human WEE1 tyrosine kinase. Science.

[147] Mueller PR, Coleman TR, Kumagai A, Dunphy WG (1995). Myt1: a membrane-associated inhibitory kinase that phosphorylates Cdc2 on both threonine-14 and tyrosine-15. Science.

[148] Välikangas T, Junttila S, Rytkönen KT, Kukkonen-Macchi A, Suomi T (2022). COVID-19-specific transcriptomic signature detectable in blood across multiple cohorts. Front Genet.

[149] Bittar C, Shrivastava S, Bhanja Chowdhury J, Rahal P, Ray RB (2013). Hepatitis C virus NS2 protein inhibits DNA damage pathway by sequestering p53 to the cytoplasm. PLoS One.

[150] Wang X, Liu Y, Li K, Hao Z (2023). Roles of p53-mediated host–virus interaction in coronavirus infection. IJMS.

[151] Kumar A, Grams TR, Bloom DC, Toth Z (2022). Signaling pathway reporter screen with SARS-CoV-2 proteins identifies nsp5 as a repressor of p53 activity. Viruses.

[152] Su M, Shi D, Xing X, Qi S, Yang D (2021). coronavirus porcine epidemic diarrhea virus nucleocapsid protein interacts with p53 to induce cell cycle arrest in s-phase and promotes viral replication. J Virol.

[153] Ma-Lauer Y, Carbajo-Lozoya J, Hein MY, Müller MA, Deng W (2016). p53 down-regulates SARS coronavirus replication and is targeted by the SARS-unique domain and PLpro via E3 ubiquitin ligase RCHY1. Proc Natl Acad Sci U S A.

[154] Jung YS, Hakem A, Hakem R, Chen X (2011). Pirh2 E3 ubiquitin ligase monoubiquitinates DNA polymerase eta to suppress translesion DNA synthesis. Mol Cell Biol.

[155] Choi M, Choi YM, An I-S, Bae S, Jung JH (2020). E3 ligase RCHY1 negatively regulates HDAC2. Biochem Biophys Res Commun.

[156] Wu H, Zeinab RA, Flores ER, Leng RP (2011). Pirh2, a ubiquitin E3 ligase, inhibits p73 transcriptional activity by promoting its ubiquitination. Mol Cancer Res.

[157] Bohgaki M, Hakem A, Halaby MJ, Bohgaki T, Li Q (2013). The E3 ligase PIRH2 polyubiquitylates CHK2 and regulates its turnover. Cell Death Differ.

[158] Laurent EMN, Sofianatos Y, Komarova A, Gimeno J-P, Tehrani PS (2020). Global BioID-based SARS-CoV-2 proteins proximal interactome unveils novel ties between viral polypeptides and host factors involved in multiple COVID19-associated mechanisms. Syst Biol.

[159] Ren H, Ma C, Peng H, Zhang B, Zhou L (2021). Micronucleus production, activation of DNA damage response and cGAS-STING signaling in syncytia induced by SARS-CoV-2 infection. Biol Direct.

[160] Basaran MM, Hazar M, Aydın M, Uzuğ G, Özdoğan İ (2023). Effects of COVID-19 disease on DNA damage, oxidative stress and immune responses. Toxics.

[161] Gain C, Song S, Angtuaco T, Satta S, Kelesidis T (2022). The role of oxidative stress in the pathogenesis of infections with coronaviruses. Front Microbiol.

[162] Suhail S, Zajac J, Fossum C, Lowater H, McCracken C (2020). Role of oxidative stress on SARS-CoV (SARS) and SARS-CoV-2 (COVID-19) infection: a review. Protein J.

[163] Youn JY, Zhang Y, Wu Y, Cannesson M, Cai H (2021). Therapeutic application of estrogen for COVID-19: attenuation of SARS-CoV-2 spike protein and IL-6 stimulated, ACE2-dependent NOX2 activation, ROS production and MCP-1 upregulation in endothelial cells. Redox Biol.

[164] Hati S, Bhattacharyya S (2020). Impact of thiol-disulfide balance on the binding of covid-19 spike protein with angiotensin-converting enzyme 2 receptor. ACS Omega.

[165] Fossum CJ, Laatsch BF, Lowater HR, Narkiewicz-Jodko AW, Lonzarich L (2022). Pre-existing oxidative stress creates a docking-ready conformation of the SARS-CoV-2 receptor-binding domain. ACS Bio Med Chem Au.

[166] Chen I-Y, Moriyama M, Chang M-F, Ichinohe T (2019). Severe acute respiratory syndrome coronavirus viroporin 3a activates the NLRP3 inflammasome. Front Microbiol.

[167] Olagnier D, Farahani E, Thyrsted J, Blay-Cadanet J, Herengt A (2020). SARS-CoV2-mediated suppression of NRF2-signaling reveals potent antiviral and anti-inflammatory activity of 4-octyl-itaconate and dimethyl fumarate. Nat Commun.

[168] Zhang S, Wang J, Wang L, Aliyari S, Cheng G (2022). SARS-CoV-2 virus NSP14 Impairs NRF2/HMOX1 activation by targeting Sirtuin 1. Cell Mol Immunol.

[169] Saheb Sharif-Askari N, Saheb Sharif-Askari F, Mdkhana B, Hussain Alsayed HA, Alsafar H (2021). Upregulation of oxidative stress gene markers during SARS-COV-2 viral infection. Free Radic Biol Med.

[170] Berquist BR, Wilson DM (2012). Pathways for repairing and tolerating the spectrum of oxidative DNA lesions. Cancer Letters.

[171] Lorente L, Martín MM, González-Rivero AF, Pérez-Cejas A, Cáceres JJ (2021). DNA and RNA oxidative damage and mortality of patients With COVID-19. Am J Med Sci.

[172] Olsen MB, Huse C, de Sousa MML, Murphy SL, Sarno A (2022). DNA repair mechanisms are activated in circulating lymphocytes of hospitalized covid-19 patients. J Inflamm Res.

[173] Bakadia BM, Boni BOO, Ahmed AAQ, Yang G (2021). The impact of oxidative stress damage induced by the environmental stressors on COVID-19. Life Sciences.

[174] Mekawy AS, Alaswad Z, Ibrahim AA, Mohamed AA, AlOkda A (2022). The consequences of viral infection on host DNA damage response: a focus on SARS-CoVs. J Genet Eng Biotechnol.

[175] Victor J, Deutsch J, Whitaker A, Lamkin EN, March A (2021). SARS-CoV-2 triggers DNA damage response in Vero E6 cells. Biochem Biophys Res Commun.

[176] Gordon DE, Hiatt J, Bouhaddou M, Rezelj VV, Ulferts S (2020). Comparative host-coronavirus protein interaction networks reveal pan-viral disease mechanisms. Science.

[177] Gordon DE, Jang GM, Bouhaddou M, Xu J, Obernier K (2020). A SARS-CoV-2 protein interaction map reveals targets for drug repurposing. Nature.

[178] Meyers JM, Ramanathan M, Shanderson RL, Beck A, Donohue L (2021). The proximal proteome of 17 SARS-CoV-2 proteins links to disrupted antiviral signaling and host translation. PLoS Pathog.

[179] Samavarchi-Tehrani P, Abdouni H, Knight JDR, Astori A, Samson R (2020). A SARS-CoV-2 – host proximity interactome. bioRxiv.

[180] Zhou Y, Liu Y, Gupta S, Paramo MI, Hou Y (2023). A comprehensive SARS-CoV-2–human protein–protein interactome reveals COVID-19 pathobiology and potential host therapeutic targets. Nat Biotechnol.

[181] Li J, Guo M, Tian X, Wang X, Yang X (2021). Virus-host interactome and proteomic survey reveal potential virulence factors influencing SARS-CoV-2 pathogenesis. Med.

[182] Liu X, Huuskonen S, Laitinen T, Redchuk T, Bogacheva M (2021). SARS-CoV-2-host proteome interactions for antiviral drug discovery. Mol Syst Biol.

[183] Li G, Tang Z, Fan W, Wang X, Huang L (2023). Atlas of interactions between SARS-CoV-2 macromolecules and host proteins. Cell Insight.

[184] Kim D-K, Weller B, Lin C-W, Sheykhkarimli D, Knapp JJ (2023). A proteome-scale map of the SARS-CoV-2-human contactome. Nat Biotechnol.

[185] Duhan N, Kaundal R (2023). HuCoPIA: an atlas of human vs. SARS-CoV-2 interactome and the comparative analysis with other *Coronaviridae* family viruses. Viruses.

[186] Nicolas E, Golemis EA, Arora S (2016). POLD1: central mediator of DNA replication and repair, and implication in cancer and other pathologies. Gene.

[187] Lipskaia L, Maisonnasse P, Fouillade C, Sencio V, Pascal Q (2022). Evidence that SARS-CoV-2 induces lung Cell senescence: potential impact on COVID-19 lung disease. Am J Respir Cell Mol Biol.

[188] D’Agnillo F, Walters K-A, Xiao Y, Sheng Z-M, Scherler K (2021). Lung epithelial and endothelial damage, loss of tissue repair, inhibition of fibrinolysis, and cellular senescence in fatal COVID-19. Sci Transl Med.

[189] Kanakkanthara A, Huntoon CJ, Hou X, Zhang M, Heinzen EP (2019). ZC3H18 specifically binds and activates the BRCA1 promoter to facilitate homologous recombination in ovarian cancer. Nat Commun.

[190] Garcia G, Jr Sharma A, Ramaiah A, Sen C, Purkayastha A (2021). Antiviral drug screen identifies DNA-damage response inhibitor as potent blocker of SARS-CoV-2 replication. Cell Reports.

[191] Sui C, Xiao T, Zhang S, Zeng H, Zheng Y (2022). SARS-CoV-2 NSP13 inhibits type I IFN production by degradation of TBK1 via p62-dependent selective autophagy. J Immunol.

[192] Vazquez C, Swanson SE, Negatu SG, Dittmar M, Miller J (2021). SARS-CoV-2 viral proteins NSP1 and NSP13 inhibit interferon activation through distinct mechanisms. PLoS One.

[193] Michelini F, Pitchiaya S, Vitelli V, Sharma S, Gioia U (2017). Damage-induced lncRNAs control the DNA damage response through interaction with DDRNAs at individual double-strand breaks. Nat Cell Biol.

[194] Pessina F, Giavazzi F, Yin Y, Gioia U, Vitelli V (2019). Functional transcription promoters at DNA double-strand breaks mediate RNA-driven phase separation of damage-response factors. Nat Cell Biol.

[195] Savastano A, Ibáñez de Opakua A, Rankovic M, Zweckstetter M (2020). Nucleocapsid protein of SARS-CoV-2 phase separates into RNA-rich polymerase-containing condensates. Nat Commun.

[196] Perdikari TM, Murthy AC, Ryan VH, Watters S, Naik MT (2020). SARS‐CoV‐2 nucleocapsid protein phase‐separates with RNA and with human hnRNPs. EMBO J.

[197] Curtin N, Bányai K, Thaventhiran J, Le Quesne J, Helyes Z (2020). Repositioning PARP inhibitors for SARS‐CoV‐2 infection(COVID‐19); a new multi‐pronged therapy for acute respiratory distress syndrome?. Br J Pharmacol.

[198] Heer CD, Sanderson DJ, Voth LS, Alhammad YMO, Schmidt MS (2020). Coronavirus infection and PARP expression dysregulate the NAD metabolome: An actionable component of innate immunity. J Biol Chem.

[199] Stone NE, Jaramillo SA, Jones AN, Vazquez AJ, Martz M (2021). Stenoparib, an inhibitor of cellular poly(ADP-Ribose) polymerase, blocks replication of the SARS-CoV-2 and HCoV-NL63 human coronaviruses *In Vitro*. mBio.

[200] Pascal JM (2018). The comings and goings of PARP-1 in response to DNA damage. DNA Repair.

[201] Langelier MF, Eisemann T, Riccio AA, Pascal JM (2018). PARP family enzymes: regulation and catalysis of the poly(ADP-ribose) posttranslational modification. Curr Opin Struct Biol.

[202] Cohen MS (2020). Interplay between compartmentalized NAD ^+^ synthesis and consumption: A focus on the PARP family. Genes Dev.

[203] Rampogu S, Jung TS, Ha MW, Lee KW (2023). Repurposing and computational design of PARP inhibitors as SARS-CoV-2 inhibitors. Sci Rep.

[204] Yang W, Kandula S, Huynh M, Greene SK, Van Wye G (2021). Estimating the infection-fatality risk of SARS-CoV-2 in New York City during the spring 2020 pandemic wave: a model-based analysis. Lancet Infect Dis.

[205] Tsilingiris D, Tentolouris A, Eleftheriadou I, Tentolouris N (2020). Telomere length, epidemiology and pathogenesis of severe COVID-19. Eur J Clin Invest.

[206] Aviv A (2020). Telomeres and COVID-19. FASEB J.

[207] Mahmoodpoor A, Sanaie S, Roudbari F, Sabzevari T, Sohrabifar N (2022). Understanding the role of telomere attrition and epigenetic signatures in COVID-19 severity. Gene.

[208] Sanchez-Vazquez R, Guío-Carrión A, Zapatero-Gaviria A, Martínez P, Blasco MA (2021). Shorter telomere lengths in patients with severe COVID-19 disease. Aging.

[209] Xu W, Zhang F, Shi Y, Chen Y, Shi B (2022). Causal association of epigenetic aging and COVID-19 severity and susceptibility: a bidirectional Mendelian randomization study. Front Med.

[210] Haridoss M, Ayyasamy L, Bagepally BS (2023). Is COVID-19 severity associated with telomere length? A systematic review and meta-analysis. Virus Genes.

[211] Benetos A, Lai T-P, Toupance S, Labat C, Verhulst S (2021). The nexus between telomere length and lymphocyte count in seniors hospitalized with COVID-19. J Gerontol A Biol Sci Med Sci.

[212] McGroder CF, Zhang D, Choudhury MA, Salvatore MM, D’Souza BM (2021). Pulmonary fibrosis 4 months after COVID-19 is associated with severity of illness and blood leucocyte telomere length. Thorax.

[213] Sepe S, Rossiello F, Cancila V, Iannelli F, Matti V (2022). DNA damage response at telomeres boosts the transcription of SARS‐CoV‐2 receptor ACE2 during aging. EMBO Reports.

[214] Tong AS, Stern JL, Sfeir A, Kartawinata M, de Lange T (2015). ATM and ATR signaling regulate the recruitment of human telomerase to telomeres. Cell Reports.

[215] d’Adda di Fagagna F (2008). Living on a break: cellular senescence as a DNA-damage response. Nat Rev Cancer.

[216] Gorgoulis V, Adams PD, Alimonti A, Bennett DC, Bischof O (2019). Cellular senescence: defining a path forward. Cell.

[217] Schmitt CA, Tchkonia T, Niedernhofer LJ, Robbins PD, Kirkland JL (2023). COVID-19 and cellular senescence. Nat Rev Immunol.

[218] Lee S, Yu Y, Trimpert J, Benthani F, Mairhofer M (2021). Virus-induced senescence is a driver and therapeutic target in COVID-19. Nature.

[219] Evangelou K, Veroutis D, Paschalaki K, Foukas PG, Lagopati N (2022). Pulmonary infection by SARS-CoV-2 induces senescence accompanied by an inflammatory phenotype in severe COVID-19: possible implications for viral mutagenesis. Eur Respir J.

[220] Mårtensson CU, Priesnitz C, Song J, Ellenrieder L, Doan KN (2019). Mitochondrial protein translocation-associated degradation. Nature.

[221] Shang C, Liu Z, Zhu Y, Lu J, Ge C (2021). SARS-CoV-2 causes mitochondrial dysfunction and mitophagy impairment. Front Microbiol.

[222] Li X, Hou P, Ma W, Wang X, Wang H (2022). SARS-CoV-2 ORF10 suppresses the antiviral innate immune response by degrading MAVS through mitophagy. Cell Mol Immunol.

[223] Perumal R, Shunmugam L, Naidoo K, Wilkins D, Garzino-Demo A (2023). Biological mechanisms underpinning the development of long COVID. iScience.

[224] Perumal R, Shunmugam L, Naidoo K, Abdool Karim SS, Wilkins D (2023). Long COVID: a review and proposed visualization of the complexity of long COVID. Front Immunol.

[225] Astin R, Banerjee A, Baker MR, Dani M, Ford E (2023). Long COVID: mechanisms, risk factors and recovery. Exp Physiol.

[226] Oronsky B, Larson C, Hammond TC, Oronsky A, Kesari S (2023). A review of persistent post-COVID syndrome (PPCS). Clin Rev Allergy Immunol.

[227] Chen B, Julg B, Mohandas S, Bradfute SB (2023). RECOVER mechanistic pathways task force. viral persistence, reactivation, and mechanisms of long COVID. Elife.

[228] Brodin P, Casari G, Townsend L, O’Farrelly C, Tancevski I (2022). Studying severe long COVID to understand post-infectious disorders beyond COVID-19. Nat Med.

[229] Stein SR, Ramelli SC, Grazioli A, Chung J-Y, Singh M (2022). SARS-CoV-2 infection and persistence in the human body and brain at autopsy. Nature.

[230] Qian Q, Fan L, Liu W, Li J, Yue J (2021). Direct evidence of active SARS-CoV-2 replication in the intestine. Clin Infect Dis.

[231] Xiang Q, Feng Z, Diao B, Tu C, Qiao Q (2021). SARS-CoV-2 induces lymphocytopenia by promoting inflammation and decimates secondary lymphoid organs. Front Immunol.

[232] Gaspar-Rodríguez A, Padilla-González A, Rivera-Toledo E (2021). Coronavirus persistence in human respiratory tract and cell culture: an overview. Braz J Infect Dis.

[233] Gaebler C, Wang Z, Lorenzi JCC, Muecksch F, Finkin S (2021). Evolution of antibody immunity to SARS-CoV-2. Nature.

[234] Li N, Wang X, Lv T (2020). Prolonged SARS-CoV-2 RNA shedding: not a rare phenomenon. J Med Virol.

[235] Chen B, Julg B, Mohandas S, Bradfute SB (2023). RECOVER mechanistic pathways task force. viral persistence, reactivation, and mechanisms of long COVID. Elife.

[236] Chen Z, Hu J, Liu L, Chen R, Wang M (2021). SARS-CoV-2 causes acute kidney injury by directly infecting renal tubules. Front Cell Dev Biol.

[237] Cevik M, Tate M, Lloyd O, Maraolo AE, Schafers J (2021). SARS-CoV-2, SARS-CoV, and MERS-CoV viral load dynamics, duration of viral shedding, and infectiousness: a systematic review and meta-analysis. Lancet Microbe.

[238] Tejerina F, Catalan P, Rodriguez-Grande C, Adan J, Rodriguez-Gonzalez C (2022). Post-COVID-19 syndrome. SARS-CoV-2 RNA detection in plasma, stool, and urine in patients with persistent symptoms after COVID-19. BMC Infect Dis.

[239] Cheung CCL, Goh D, Lim X, Tien TZ, Lim JCT (2022). Residual SARS-CoV-2 viral antigens detected in GI and hepatic tissues from five recovered patients with COVID-19. Gut.

[240] Zhang L (2021). Reverse-transcribed SARS-CoV-2 RNA can integrate into the genome of cultured human cells and can be expressed in patient-derived tissues. Proc Natl Acad Sci USA.

[241] Briggs E, Ward W, Rey S, Law D, Nelson K (2021). Assessment of potential SARS-CoV-2 virus integration into human genome reveals no significant impact on RT-qPCR COVID-19 testing. Proc Natl Acad Sci U S A.

[242] Parry R, Gifford RJ, Lytras S, Ray SC, Coin LJM (2021). No evidence of SARS-CoV-2 reverse transcription and integration as the origin of chimeric transcripts in patient tissues. Proc Natl Acad Sci U S A.

[243] Mahmood S, Kawanaka M, Kamei A, Izumi A, Nakata K (2004). Immunohistochemical evaluation of oxidative stress markers in chronic hepatitis C. Antioxid Redox Signal.

[244] Konishi M, Iwasa M, Araki J, Kobayashi Y, Katsuki A (2006). Increased lipid peroxidation in patients with non-alcoholic fatty liver disease and chronic hepatitis C as measured by the plasma level of 8-isoprostane. J Gastroenterol Hepatol.

[245] Farinati F, Cardin R, Bortolami M, Burra P, Russo FP (2007). Hepatitis C virus: from oxygen free radicals to hepatocellular carcinoma. J Viral Hepat.

[246] Machida K, Cheng KT-N, Sung VM-H, Shimodaira S, Lindsay KL (2004). Hepatitis C virus induces a mutator phenotype: enhanced mutations of immunoglobulin and protooncogenes. Proc Natl Acad Sci U S A.

[247] Machida K, Cheng KT-H, Sung VM-H, Lee KJ, Levine AM (2004). Hepatitis C virus infection activates the immunologic (type II) isoform of nitric oxide synthase and thereby enhances DNA damage and mutations of cellular genes. J Virol.

[248] Machida T, Takahashi T, Itoh T, Hirayama M, Morita T (2007). Reactive lymphoid hyperplasia of the liver: a case report and review of literature. World J Gastroenterol.

[249] Machida K, Cheng KT-H, Lai C-K, Jeng K-S, Sung VM-H (2006). Hepatitis C virus triggers mitochondrial permeability transition with production of reactive oxygen species, leading to DNA damage and STAT3 activation. J Virol.

[250] Machida K, Cheng KTH, Sung VM-H, Levine AM, Foung S (2006). Hepatitis C virus induces toll-like receptor 4 expression, leading to enhanced production of beta interferon and interleukin-6. J Virol.

[251] Higgs MR, Chouteau P, Lerat H (2014). “Liver let die”: oxidative DNA damage and hepatotropic viruses. J Gen Virol.

[252] Nikitin PA, Luftig MA (2011). At a crossroads: human DNA tumor viruses and the host DNA damage response. Future Virol.

[253] Tardivat Y, Sosnowski P, Tidu A, Westhof E, Eriani G (2023). SARS-CoV-2 NSP1 induces mRNA cleavages on the ribosome. Nucleic Acids Res.

[254] Schubert K, Karousis ED, Jomaa A, Scaiola A, Echeverria B (2020). SARS-CoV-2 Nsp1 binds the ribosomal mRNA channel to inhibit translation. Nat Struct Mol Biol.

[255] Thoms M, Buschauer R, Ameismeier M, Koepke L, Denk T (2020). Structural basis for translational shutdown and immune evasion by the Nsp1 protein of SARS-CoV-2. Science.

[256] Xu Z, Choi J-H, Dai DL, Luo J, Ladak RJ (2022). SARS-CoV-2 impairs interferon production via NSP2-induced repression of mRNA translation. Proc Natl Acad Sci U S A.

[257] Gupta M, Azumaya CM, Moritz M, Pourmal S, Diallo A (2021). CryoEM and AI reveal a structure of SARS-CoV-2 Nsp2, a multifunctional protein involved in key host processes. bioRxiv.

[258] Li P, Xue B, Schnicker NJ, Wong L-YR, Meyerholz DK (2023). Nsp3-N interactions are critical for SARS-CoV-2 fitness and virulence. Proc Natl Acad Sci USA.

[259] Armstrong LA, Lange SM, Dee Cesare V, Matthews SP, Nirujogi RS (2021). Biochemical characterization of protease activity of Nsp3 from SARS-CoV-2 and its inhibition by nanobodies. PLoS One.

[260] Angelini MM, Akhlaghpour M, Neuman BW, Buchmeier MJ (2013). Severe acute respiratory syndrome coronavirus nonstructural proteins 3, 4, and 6 induce double-membrane vesicles. mBio.

[261] Liu Y, Qin C, Rao Y, Ngo C, Feng JJ (2021). SARS-CoV-2 Nsp5 demonstrates two distinct mechanisms targeting RIG-I and MAVS to evade the innate immune response. mBio.

[262] Ricciardi S, Guarino AM, Giaquinto L, Polishchuk EV, Santoro M (2022). The role of NSP6 in the biogenesis of the SARS-CoV-2 replication organelle. Nature.

[263] Hillen HS, Kokic G, Farnung L, Dienemann C, Tegunov D (2020). Structure of replicating SARS-CoV-2 polymerase. Nature.

[264] Wang Q, Wu J, Wang H, Gao Y, Liu Q (2020). Structural basis for RNA replication by the SARS-CoV-2 polymerase. Cell.

[265] Deng J, Zheng Y, Zheng S-N, Nan M-L, Han L (2023). SARS-CoV-2 NSP7 inhibits type I and III IFN production by targeting the RIG-I/MDA5, TRIF, and STING signaling pathways. J Med Virol.

[266] Deng J, Zheng S-N, Xiao Y, Nan M-L, Zhang J (2023). SARS-CoV-2 NSP8 suppresses type I and III IFN responses by modulating the RIG-I/MDA5, TRIF, and STING signaling pathways. J Med Virol.

[267] Park GJ, Osinski A, Hernandez G, Eitson JL, Majumdar A (2022). The mechanism of RNA capping by SARS-CoV-2. Nature.

[268] Xiang JS, Mueller JR, Luo E-C, Yee BA, Schafer D (2022). Discovery and functional interrogation of SARS-CoV-2 protein-RNA interactions. bioRxiv.

[269] El-Kamand S, Du Plessis M-D, Breen N, Johnson L, Beard S (2022). A distinct ssDNA/RNA binding interface in the Nsp9 protein from SARS-CoV-2. Proteins.

[270] Riccio AA, Sullivan ED, Copeland WC (2022). Activation of the SARS-CoV-2 NSP14 3’-5’ exoribonuclease by NSP10 and response to antiviral inhibitors. J Biol Chem.

[271] Klima M, Khalili Yazdi A, Li F, Chau I, Hajian T (2022). Crystal structure of SARS-CoV-2 nsp10-nsp16 in complex with small molecule inhibitors, SS148 and WZ16. Protein Sci.

[272] Gadhave K, Kumar P, Kumar A, Bhardwaj T, Garg N (2021). Conformational dynamics of 13 amino acids long NSP11 of SARS-CoV-2 under membrane mimetics and different solvent conditions. Microb Pathog.

[273] Yin W, Mao C, Luan X, Shen D-D, Shen Q (2020). Structural basis for inhibition of the RNA-dependent RNA polymerase from SARS-CoV-2 by remdesivir. Science.

[274] Yuen C-K, Lam J-Y, Wong W-M, Mak L-F, Wang X (2020). SARS-CoV-2 nsp13, nsp14, nsp15 and orf6 function as potent interferon antagonists. Emerg Microbes Infect.

[275] Sui C, Xiao T, Zhang S, Zeng H, Zheng Y (2022). SARS-CoV-2 NSP13 inhibits type I IFN production by degradation of TBK1 via p62-dependent selective autophagy. J Immunol.

[276] Imprachim N, Yosaatmadja Y, Newman JA (2023). Crystal structures and fragment screening of SARS-CoV-2 NSP14 reveal details of exoribonuclease activation and mRNA capping and provide starting points for antiviral drug development. Nucleic Acids Res.

[277] Pan R, Kindler E, Cao L, Zhou Y, Zhang Z (2022). N7-methylation of the coronavirus RNA cap is required for maximal virulence by preventing innate immune recognition. mBio.

[278] Wilson IM, Frazier MN, Li JL, Randall TA, Stanley RE (2022). Biochemical characterization of emerging SARS-CoV-2 Nsp15 endoribonuclease variants. J Mol Biol.

[279] Russ A, Wittmann S, Tsukamoto Y, Herrmann A, Deutschmann J (2022). Nsp16 shields SARS-CoV-2 from efficient MDA5 sensing and IFIT1-mediated restriction. EMBO Rep.

[280] Vithani N, Ward MD, Zimmerman MI, Novak B, Borowsky JH (2021). SARS-CoV-2 Nsp16 activation mechanism and a cryptic pocket with pan-coronavirus antiviral potential. Biophys J.

[281] Zheng M, Karki R, Williams EP, Yang D, Fitzpatrick E (2021). TLR2 senses the SARS-CoV-2 envelope protein to produce inflammatory cytokines. Nat Immunol.

[282] Oh SJ, Shin OS (2021). SARS-CoV-2 nucleocapsid protein targets RIG-I-like receptor pathways to inhibit the induction of interferon response. Cells.

[283] Chen K, Xiao F, Hu D, Ge W, Tian M (2020). SARS-CoV-2 nucleocapsid protein interacts with RIG-I and represses RIG-mediated IFN-β production. Viruses.

[284] Guruprasad L (2021). Human coronavirus spike protein-host receptor recognition. Prog Biophys Mol Biol.

[285] Wang Q, Zhang Y, Wu L, Niu S, Song C (2020). Structural and functional basis of SARS-CoV-2 entry by using human ACE2. Cell.

[286] Xu H, Akinyemi IA, Chitre SA, Loeb JC, Lednicky JA (2022). SARS-CoV-2 viroporin encoded by ORF3a triggers the NLRP3 inflammatory pathway. Virology.

[287] Arshad N, Laurent-Rolle M, Ahmed WS, Hsu JC-C, Mitchell SM (2023). SARS-CoV-2 accessory proteins ORF7a and ORF3a use distinct mechanisms to down-regulate MHC-I surface expression. Proc Natl Acad Sci USA.

[288] Castaño-Rodriguez C, Honrubia JM, Gutiérrez-Álvarez J, DeDiego ML, Nieto-Torres JL (2018). Role of severe acute respiratory syndrome coronavirus viroporins E, 3a, and 8a in replication and pathogenesis. mBio.

[289] Kern DM, Sorum B, Mali SS, Hoel CM, Sridharan S (2021). Cryo-EM structure of SARS-CoV-2 ORF3a in lipid nanodiscs. Nat Struct Mol Biol.

[290] Siu K, Yuen K, Castano‐Rodriguez C, Ye Z, Yeung M (2019). Severe acute respiratory syndrome coronavirus ORF3a protein activates the NLRP3 inflammasome by promoting TRAF3‐dependent ubiquitination of ASC. FASEB J.

[291] Nie Y, Mou L, Long Q, Deng D, Hu R (2023). SARS-CoV-2 ORF3a positively regulates NF-κB activity by enhancing IKKβ-NEMO interaction. Virus Res.

[292] Ren Y, Shu T, Wu D, Mu J, Wang C (2020). The ORF3a protein of SARS-CoV-2 induces apoptosis in cells. Cell Mol Immunol.

[293] Konno Y, Kimura I, Uriu K, Fukushi M, Irie T (2020). SARS-CoV-2 ORF3b is a potent interferon antagonist whose activity is increased by a naturally occurring elongation variant. Cell Rep.

[294] Miorin L, Kehrer T, Sanchez-Aparicio MT, Zhang K, Cohen P (2020). SARS-CoV-2 Orf6 hijacks Nup98 to block STAT nuclear import and antagonize interferon signaling. Proc Natl Acad Sci U S A.

[295] Addetia A, Lieberman NAP, Phung Q, Hsiang T-Y, Xie H (2021). SARS-CoV-2 ORF6 disrupts bidirectional nucleocytoplasmic transport through interactions with Rae1 and Nup98. mBio.

[296] Lee J-G, Huang W, Lee H, van de Leemput J, Kane MA (2021). Characterization of SARS-CoV-2 proteins reveals Orf6 pathogenicity, subcellular localization, host interactions and attenuation by selinexor. Cell Biosci.

[297] Su CM, Wang L, Yoo D (2021). Activation of NF-κB and induction of proinflammatory cytokine expressions mediated by ORF7a protein of SARS-CoV-2. Sci Rep.

[298] Hou P, Wang X, Wang H, Wang T, Yu Z (2023). The ORF7a protein of SARS-CoV-2 initiates autophagy and limits autophagosome-lysosome fusion via degradation of SNAP29 to promote virus replication. Autophagy.

[299] García-García T, Fernández-Rodríguez R, Redondo N, de Lucas-Rius A, Zaldívar-López S (2022). Impairment of antiviral immune response and disruption of cellular functions by SARS-CoV-2 ORF7a and ORF7b. iScience.

[300] Flower TG, Buffalo CZ, Hooy RM, Allaire M, Ren X (2021). Structure of SARS-CoV-2 ORF8, a rapidly evolving immune evasion protein. Proc Natl Acad Sci U S A.

[301] Wu X, Xia T, Shin W-J, Yu K-M, Jung W (2022). Viral mimicry of interleukin-17A by SARS-CoV-2 ORF8. mBio.

[302] Lin X, Fu B, Yin S, Li Z, Liu H (2021). ORF8 contributes to cytokine storm during SARS-CoV-2 infection by activating IL-17 pathway. iScience.

[303] Zhang Y, Chen Y, Li Y, Huang F, Luo B (2021). The ORF8 protein of SARS-CoV-2 mediates immune evasion through down-regulating MHC-Ι. Proc Natl Acad Sci USA.

[304] Han L, Zhuang M-W, Deng J, Zheng Y, Zhang J (2021). SARS-CoV-2 ORF9b antagonizes type I and III interferons by targeting multiple components of the RIG-I/MDA-5-MAVS, TLR3-TRIF, and cGAS-STING signaling pathways. J Med Virol.

[305] Wu J, Shi Y, Pan X, Wu S, Hou R (2021). SARS-CoV-2 ORF9b inhibits RIG-I-MAVS antiviral signaling by interrupting K63-linked ubiquitination of NEMO. Cell Rep.

[306] Thorne LG, Bouhaddou M, Reuschl A-K, Zuliani-Alvarez L, Polacco B (2022). Evolution of enhanced innate immune evasion by SARS-CoV-2. Nature.

[307] Ayinde KS, Pinheiro GMS, Ramos CHI (2022). Binding of SARS-CoV-2 protein ORF9b to mitochondrial translocase TOM70 prevents its interaction with chaperone HSP90. Biochimie.

[308] Gao X, Zhu K, Qin B, Olieric V, Wang M (2021). Crystal structure of SARS-CoV-2 Orf9b in complex with human TOM70 suggests unusual virus-host interactions. Nat Commun.

[309] Jiang H, Zhang H, Meng Q, Xie J, Li Y (2020). SARS-CoV-2 Orf9b suppresses type I interferon responses by targeting TOM70. Cell Mol Immunol.

[310] Dominguez Andres A, Feng Y, Campos AR, Yin J, Yang C-C (2020). SARS-CoV-2 ORF9c is a membrane-associated protein that suppresses antiviral responses in cells. bioRxiv.

[311] Han L, Zheng Y, Deng J, Nan M-L, Xiao Y (2022). SARS-CoV-2 ORF10 antagonizes STING-dependent interferon activation and autophagy. J Med Virol.

